# Mitochondrial Metabolic Biomarkers in Periodontitis: Discovery and Clinical Validation

**DOI:** 10.1016/j.identj.2026.109682

**Published:** 2026-06-13

**Authors:** Jiahua Wu, Juan Wu, Yubing Hong, Lijia Rao, Mu Chen

**Affiliations:** Department of Stomatology, Shenzhen Nanshan People's Hospital, Shenzhen, Guangdong, 518000, China

**Keywords:** Periodontitis, Mitochondrial metabolism, Biomarkers, Machine learning, Single-cell RNA sequencing, Animal model

## Abstract

**Introduction and Aims:**

Mitochondrial metabolic dysregulation is associated with periodontitis (PD); however, related biomarkers remain unclear. In this study, we identified key mitochondrial metabolism-related biomarkers in PD through integrated bioinformatics technology.

**Methods:**

Transcriptomic and single-cell RNA sequencing data from the Gene Expression Omnibus database were analysed using machine learning to identify biomarkers. A diagnostic nomogram was constructed. Immune infiltration, drug prediction, and T-cell dynamic expression were assessed. Putative biomarkers were validated in clinical samples and a rat PD model by reverse transcription-quantitative polymerase chain reaction, immunohistochemistry, and Western blot analysis.

**Results:**

Six biomarkers were identified, which included ENTPD1, CYP24A1, ADA, TDO2 (upregulated), OSBPL6, and NUDT15 (downregulated). The nomogram exhibited excellent diagnostic efficacy (area under the curve [AUC] = 0.954). The biomarkers correlated with plasma cells and resting dendritic cells. Single-cell analysis revealed dynamic expression during T-cell differentiation. CTA018 exhibited strong binding to CYP24A1. Five biomarkers were validated in clinical samples, and the upregulation of ENTPD1, CYP24A1, and TDO2 protein and messenger RNA was confirmed in a rat model and correlated with PD progression.

**Conclusion:**

ENTPD1, CYP24A1, and TDO2 are mitochondrial metabolism-related biomarkers with high diagnostic potential in PD and targets for therapy.

**Clinical Relevance:**

The identified biomarkers, particularly ENTPD1, CYP24A1, and TDO2, have strong diagnostic value. The nomogram contributed to PD risk stratification. Associations with immune infiltration and drug sensitivity may guide immunotherapy and targeted therapy.

## Highlights

• Six mitochondrial metabolism-related biomarkers were identified for periodontitis diagnosis.
• A 6-gene nomogram achieved excellent diagnostic performance for periodontitis.
• Single-cell analysis identified T cells as key cells in periodontitis progression.
• CTA018 showed the strongest predicted binding affinity with CYP24A1.
• Clinical samples and rat models validated ENTPD1, CYP24A1, and TDO2.

## Introduction

Periodontitis (PD) is a chronic inflammatory disease of the tooth-supporting tissues that poses significant risks to oral health. It affects nearly half of the global population and is the sixth most prevalent chronic disease worldwide.^1^, ^2^, ^3^, ^4^ PD is a multifactorial disease primarily caused by microbial factors, although host immune responses, local factors (eg, plaque accumulation), systemic conditions (eg, diabetes), and genetic predisposition can influence its pathogenesis, progression, and outcomes.^5^ PD manifests as inflammation of the connective tissues and alveolar bone resorption, which ultimately leads to tooth mobility and loss.^6^ Current therapies include nonsurgical treatment (scaling and root planing), surgical intervention (eg, flap surgery), and pharmacologic management (eg, antibiotics).^7^ Despite these approaches and preventive measures, the prevalence of PD remains high.^8^ Treatment efficacy is often suboptimal in severe cases or in patients with systemic comorbidities. Recurrence may occur if plaque control is inadequate posttreatment.^9^ This is closely associated with the difficulty in early diagnosis, as current clinical diagnosis primarily relies on traditional parameters, such as probing depth and clinical attachment level. There is a lack of objective and sensitive molecular biomarkers, which results in the disease being diagnosed at an advanced stage. Therefore, improving the early and accurate diagnosis of PD, reducing its incidence and severity, and developing effective treatment strategies depend on the identification of reliable molecular biomarkers, establishing objective diagnostic models, and elucidating their underlying pathogenic mechanisms.

Mitochondrial metabolism is an important component of the cellular metabolic network.^10^ By generating adenosine triphosphate (ATP), mitochondria provide the primary energy source for all cellular activities.^11^ They also play important roles in diverse cellular processes, including reactive oxygen species (ROS) regulation, apoptosis modulation, calcium ion homeostasis, and signal transduction.^12^ Mitochondrial metabolism is associated with various chronic diseases, including PD.^13^^,^^14^ Periodontal pathogens can trigger the host immune response, resulting in excessive ROS production, which can trigger apoptosis, exacerbate inflammation, and promote alveolar bone loss.^15^^,^^16^ Mitochondria represent the primary source of ROS production.^17^ Moreover, pathogens can disrupt the clearance of damaged mitochondria by interfering with the expression of mitophagy-related genes, thereby amplifying the inflammatory response in macrophages.^18^ An imbalance in mitochondrial metabolism, the excessive production of mitochondrial ROS, dysregulation of mitochondrial biogenesis and dynamics, impaired mitophagy, and mitochondrial DNA damage can all affect the initiation and progression of PD.^19^, ^20^, ^21^, ^22^ Currently, the underlying mechanisms of mitochondrial metabolism-related genes (MMRGs) in PD are unclear. Therefore, understanding mitochondrial metabolism is important for identifying novel therapeutic targets and treatment strategies.

Recently, novel proinflammatory macrophage subtypes have been identified, leading to the development of a high-performance 5-gene diagnostic model by Chen et al.^23^ Similarly, Zhang et al^24^ performed an integrative analysis and identified shared biomarkers linking PD to oral squamous cell carcinoma, including SERPINA1. These studies highlight the potential role of bioinformatics combined with machine learning for identifying cellular heterogeneity in periodontal disease and diagnostic signatures. Based on these findings, we identified biomarkers related to mitochondrial metabolism in PD through a bioinformatics analysis of RNA sequencing (RNA-seq) data from public databases. A biomarker-based diagnostic model for PD was constructed, followed by a comprehensive analysis of the biological functions and molecular mechanisms associated with the selected biomarkers. Subsequently, drug target prediction analysis was performed. Our results provide insight into the mechanistic role of mitochondrial metabolism in PD pathogenesis, early clinical diagnosis of patients with periodontal disease, and the development of personalised immunotherapeutic strategies.

## Materials and methods

### Data acquisition

The transcriptome data used in this study were obtained from the Gene Expression Omnibus database (https://www.ncbi.nlm.nih.gov/geo/). GSE16134 (GPL570 platform) and GSE10334 (GPL570 platform) represent classic bulk RNA-seq datasets. The GSE16134 dataset comprises 241 PD and 69 healthy gingival tissue samples. GSE10334 contains 183 PD and 64 healthy samples, whereas GSE164241 (platform: GPL18573) is a single-cell RNA-seq (scRNA-seq) dataset with 8 PD and 13 healthy gingival tissue samples. The samples were collected from human participants.

In addition, we obtained data for 1234 MMRGs from Meng et al^25^ (Supplementary Table 1). This gene list was retrieved and compiled by the original authors based on the Molecular Signatures Database (MSigDB).

### Identification and functional analyses of candidate genes

Using the limma package (v 3.56.2),^26^ differential gene expression analysis between the PD and control groups was done on the GSE16134 dataset to identify differentially expressed genes (DEGs) (adjusted *P* < .05, |log_2_fold change| > 0.5). The results were subsequently visualised using ggplot2 (v 3.5.1)^27^ and pheatmap (v 1.0.12).^28^

Candidate genes associated with mitochondrial metabolism in PD were identified by intersecting the DEGs and MMRGs using the ggvenn package (v 0.1.10).^29^ Functional enrichment analysis of these candidates was conducted using Gene Ontology (GO) and Kyoto Encyclopedia of Genes and Genomes (KEGG) through the clusterProfiler package (v 4.15.0.003).^30^ A protein-protein interaction network was constructed using the Search Tool for the Retrieval of Interacting Genes/Proteins (STRING) database (https://string-db.org/) and visualised with Cytoscape software (v 3.9.1)^31^ using a confidence score threshold of >0.4.

### Recognition and diagnostic value analysis of the biomarkers

Candidate genes from the GSE16134 dataset were initially analysed by 2 machine learning algorithms to identify mitochondrial metabolism-related biomarkers in PD. Specifically, the glmnet package (v 4.1.8)^32^ was used to construct a 10-fold cross-validated least absolute shrinkage and selection operator (LASSO) model to derive LASSO signature genes (α = 1). In addition, the caret package (v 6.0.94)^33^ was used for a 10-fold cross-validated support vector machine–recursive feature elimination (SVM-RFE) analysis to identify SVM-RFE signature genes, which were then intersected with the LASSO signature genes using ggvenn (v 0.1.10). Receiver operating characteristic (ROC) curves for the GSE16134 and GSE10334 genes were plotted using the pROC package (v 1.18.5).^34^ Genes with area under the curve (AUC) values >0.7 were considered candidate biomarkers. Differences in candidate biomarker expression across groups in both datasets were visualised and evaluated using the Wilcoxon rank-sum test (*P* < .05). Genes showing consistent expression trends and significant differences across both datasets were defined as mitochondrial metabolism-related PD biomarkers.

To assess their diagnostic value, a nomogram model integrating all mitochondrial metabolism-related biomarkers was established using the rms package (v 6.8.1)^35^ based on their expression in GSE16134 to predict PD incidence. Model performance was assessed using an ROC curve (AUC > 0.7), calibration curve (Hosmer-Lemeshow test, *P* > .05), and decision curve analysis (DCA) using regplot (v 1.1),^36^ pROC (v 1.18.5), and ggDCA (v 1.1),^37^ respectively.

To further assess the generalisation ability of the above diagnostic model using independent data, GSE10334 was used as an external validation cohort. The expression values of the 6 biomarkers for each sample in GSE10334 were substituted into the diagnostic model, which was constructed based on the GSE16134 training set, and the predicted risk score for each sample was calculated. Based on the predicted scores and the actual disease status, a calibration curve, an ROC curve, and a DCA decision curve were plotted to determine the calibration, discrimination, and net clinical benefit of the model. The R packages used were consistent with those used in the training set evaluation.

### Correlation analysis of the biomarkers

In the GSE16134 dataset, a Spearman correlation analysis between metabolism-related biomarkers was performed using the psych package (v 2.4.6.26)^38^ (|correlation coefficients [cor]| > 0.3, *P* < .05).

### Gene set enrichment analysis

Gene set enrichment analysis (GSEA) was conducted on the GSE16134 dataset to identify signalling pathways associated with the mitochondrial metabolism-related biomarkers in PD. The Spearman correlation between the biomarkers and all other genes was performed using the cor function in the stats package (v 4.4.1)^39^ with the genes sorted in descending order. Next, GSEA was performed using clusterProfiler (v 4.15.0.003) for each biomarker (|normalised enrichment score| > 1, adjusted *P* < .05) against the Hallmark gene sets from the MSigDB.

### Immune infiltration analysis

CIBERSORT (version 0.1.0)^40^ was used to assess the differential immune microenvironment between the PD group and the control group from the GSE16134 dataset, which estimated the infiltration abundance of 22 immune cell types.^41^ The analysis was run in relative mode, and the relative proportion of each immune cell type in each sample was determined. To evaluate the reliability of the deconvolution results, 100 permutation tests were performed for each sample. Only the samples with a permutation value <0.05 were considered to have statistically credible estimates of immune cell composition and were included in subsequent analyses. Based on the results, the Wilcoxon rank-sum test was used to screen for immune cell types that were differentially infiltrated between the PD and control groups (*P* < .05). Spearman correlation analysis was then used to evaluate the correlations among the differential immune cell types and between these cell types and the mitochondria‑related metabolic biomarkers (|cor| > 0.3, *P* < .05). The above analyses were conducted using the psych package (v 2.4.6.26).^38^

### Small-molecule compound prediction and molecular docking

Clues for the treatment of PD were provided by predicting small-molecule compounds that target mitochondrial metabolism-related biomarkers through the Drug-Gene Interaction Database (https://dgidb.org/). An interaction network of small-molecule compounds and biomarkers was visualised using Cytoscape (v 3.9.1), and compounds with the highest interaction scores for each biomarker were considered key small-molecule compounds. Next, the predicted small-molecule compounds and corresponding biomarkers were subjected to molecular docking. Specifically, 3-dimensional structures of the molecular receptors (biomarkers) and ligands (small-molecule compounds) were acquired from the Universal Protein Resource (UniProt) (http://www.uniprot.org/) and PubChem (https://pubchem.ncbi.nlm.nih.gov/) databases, respectively. The CB-Dock platform (https://cadd.labshare.cn/cb-dock2/php/blinddock.php) was used to facilitate the docking processes, and the binding energies were calculated. If the binding energy was less than −5.0 kcal/mol, the key small-molecule compound was considered to exhibit strong binding affinity with the target protein. The optimal complexes of key small-molecule compounds and the corresponding biomarkers were visualised using PyMol software (v 3.0.3)^42^ and highlight the binding sites.

### Molecular regulatory network construction

To identify the regulatory mechanisms of the mitochondrial metabolism-related biomarkers in PD, microRNAs (miRNAs) targeting the biomarkers were predicted using Miranda (http://mirtoolsgallery.tech/mirtoolsgallery/node/1055) and miRDB (https://mirdb.org/). The miRNAs predicted by both databases were used to predict the corresponding long noncoding RNAs (lncRNAs) in the starBase database (https://starbase.sysu.edu.cn/), thus enabling the construction of an lncRNA-miRNA–messenger RNA (mRNA) regulatory network.

### Cell-type profiling and key cell-type identification using scRNA-seq analysis

A detailed analysis of scRNA-seq data from GSE164241 was conducted to explore the cellular mechanisms underlying PD progression. Cell composition was characterised using Seurat (v 5.3.0)^43^ for preprocessing with stringent Quality Control. The genes expressed in ≥3 cells and cells with RNA feature count <6000, total RNA count <20,000, and a mitochondrial RNA percentage <25% were retained. Because the data originated from multiple samples, to reduce the effect of technical differences between samples during downstream analyses, we used the canonical correlation analysis–based integration workflow in Seurat v5 for batch effect correction. The data from each sample were first normalised using LogNormalize, and 2000 highly variable genes were selected (FindVariableFeatures). Principal component analysis (PCA) dimensionality reduction was subsequently performed based on these highly variable genes, and anchor cells across samples (anchors, k.anchor = 20) were identified using the canonical correlation analysis method to achieve data alignment and integration. PCA was conducted using the RunPCA function to identify potential outlier samples. The ElbowPlot function generated an elbow plot showing the variance explained by each principal component (PC), with statistical significance evaluated with JackStraw. PCs were selected based on the elbow plot and PCA permutation test (*P* < .05). Cell clustering was done using FindNeighbors and FindClusters (resolution = 0.3) and visualised with uniform manifold approximation and projection. Cell-type annotation was based on published marker genes^44^ and CellMarker 2.0 (http://117.50.127.228/CellMarker/), with marker expression illustrated using DotPlot. Cell-type proportions were calculated for PD and control groups and compared using the Wilcoxon rank-sum test. Notably, the key cell type was selected based on its high abundance in PD and a significant proportion difference between groups (*P* < .05). The distribution and expression of mitochondrial metabolism-related biomarkers among the distinct cell types were illustrated by the FeaturePlot and DotPlot functions, respectively.

### Cell function and cell-cell communication analyses

Functional analysis of all cell types identified in the GSE164241 dataset was conducted using the analyse_sc_clusters function in the ReactomeGSA package (v 1.16.1)^45^ to reveal their potential biological functions in the PD and control groups. The top 10 most differentially expressed pathways across cell types were visualised using the pathways function.

The CellChat package (v 2.2.0)^46^ was used to assess the number and strength of the intercellular interactions among all cell types in the PD and control groups within GSE164241 and to analyse ligand-receptor (L-R) pairs driving intercellular signalling (*P* < .05).

### Cell trajectory analysis

Within GSE164241, a cell trajectory analysis was performed using Monocle (v 2.30.1)^47^ to simulate differentiation of the key cell type. The expression patterns of the mitochondrial metabolism-related biomarkers during this process were also analysed.

### Reverse transcription-quantitative polymerase chain reaction

To validate the expression of mitochondrial metabolism-related biomarkers in clinical specimens, total RNA was extracted from 5 PD tissue samples and 5 control samples using TRIzol reagent (R401-01; Ambion). The samples were collected at the Shenzhen Nanshan People’s Hospital. Complementary DNA was synthesised using the Hifair III 1st Strand cDNA Synthesis SuperMix for qPCR kit (11141ES60; Yisheng), and reverse transcription-quantitative polymerase chain reaction (RT-qPCR) was conducted using 2× Universal Blue SYBR Green qPCR Master Mix (G3326-05; Servicebio). The primer sequences for the biomarkers and GAPDH are listed in Supplementary Table 2 along with reaction conditions. Relative expression was calculated using the 2^−ΔΔCт^ method. Group differences were assessed using a *t* test (*P* < .05) and visualised with GraphPad Prism 5 software (v 8.0; GraphPad Software).^48^ Ethical approval was granted by the Ethics Committee of Shenzhen Nanshan People’s Hospital (ky-2025-011101).

To determine the statistical reliability of the RT-qPCR differential analysis based on the clinical sample size, a statistical power analysis using a 2-sided independent sample *t* test was performed. Cohen’s *d* effect sizes were calculated based on the mean and standard deviation of each gene in the PD and control groups. Based on a significance level α = 0.05 and a sample size of 5 per group, the statistical power for each gene was calculated using the pwr.t.test function in the pwr package of R. A statistical power ≥0.80 was used as the reference threshold for detecting differences between groups.

### Animal model development and sample collection

Six male Sprague-Dawley rats (6-8 weeks old, 300-320 g) were obtained from Suzhou Xishan Biotechnology (Production License No. SCXK [Jing] 2024-0001) and housed under standard conditions (22 °C to 25 °C, 50%-60% humidity, 12-hour light/dark cycle) with free access to food and water. After the rats had acclimated to their environment, animal models were established by randomly assigning the rats into 2 groups: 3 rats were ligated around the left maxillary second molar using 5-0 silk suture (the suture was gently inserted into the interdental space and knotted on the palatal side), which was designated the PD group, and the remaining 3 rats received no suture ligation and were designated the control group. They were housed continuously for 7 days, and the ligation sutures of the PD group rats remained intact without falling off. Body weight was monitored throughout the study. At the end of the experimental cycle, the rats were euthanised by cervical dislocation following inhalation anaesthesia. The maxilla, including periodontal tissues, teeth, and alveolar bone, was collected from each rat for further analysis. The right gingival tissue was stored at −80 °C, and the left gingival tissue and alveolar bone were preserved in 4% paraformaldehyde. Gross morphologic observations of the maxillae were performed. All animal experiments were approved by the Ethics Committee of Shenzhen Nanshan People’s Hospital (ky-2025-011101) and conducted in strict accordance with ethical guidelines for laboratory animal use.

### Haematoxylin and eosin and immunohistochemical staining

Haematoxylin and eosin staining was done to assess pathologic differences in the gingival tissues (preserved in 4% paraformaldehyde) between the PD and control rats. Tissue sections were baked at 64 °C for 1 hour, deparaffinised in xylene, rehydrated through a graded ethanol series, stained with haematoxylin, differentiated, and stained with eosin for 5 to 10 seconds. The samples were then dehydrated, cleared in xylene, mounted with neutral resin, and scanned using a slide scanner (SQS-12P; Shenzhen Johnson Technology) for whole-slide imaging. Differences in the percentage of inflammatory cells and the area of inflammation (μm^2^) between the control and PD groups were analysed. Intergroup differences were assessed using GraphPad Prism software (v 10.1.2) with the *t* test (*P* < .05).

Immunohistochemistry (IHC) was done to evaluate protein expression of the mitochondrial metabolism-related biomarkers in gingival tissues (4% paraformaldehyde) in the control and PD rats. Sections were baked (64 °C, 1 hour), deparaffinised in xylene, and rehydrated through a graded ethanol series, followed by antigen retrieval in citrate buffer (pressure cooker, 3 minutes). Endogenous peroxidase was quenched with 3% H_2_O_2_, and nonspecific binding was blocked with 5% bovine serum albumin (BSA). Primary antibodies (anti-ENTPD1, GB-111582-50, 1:200; anti-CYP24A1, GB-113873-50, 1:200; anti-TDO2, GB-113244-50, 1:200; Servicebio) in 2% BSA were applied overnight at 4 °C, followed by incubation at 37 °C for 40 minutes. Secondary antibodies (horseradish peroxidase [HRP]–conjugated goat anti-rabbit IgG, GB23303, 1:3000; goat anti-rat IgG, GB23301, 1:5000) were applied and incubated at 37 °C for 30 minutes, visualised with Diaminobenzidine, and counterstained with haematoxylin. The slides were dehydrated, cleared, mounted, and scanned. Image analysis was performed using Image-Pro Plus (v 4.5; Media Cybernetics, Inc., Rockville, MD, USA) and ImageJ (v 1.43; National Institutes of Health) software.^49^ Intergroup differences were assessed using GraphPad Prism software (v 10.1.2) and a t-test (*P* < .05).

### Western blot and RT-qPCR analyses

The expression of the mitochondrial metabolism-related biomarkers was analysed in the gingival tissues (stored at −80 °C) of the PD and control group rats by Western blot (WB) analysis. Total protein was extracted from the tissues using RIPA lysis buffer (G2002-30ML; Servicebio), and the protein concentration was determined using a BCA protein assay kit (P0009; Beyotime). The samples were denatured in 5× sample buffer (boiling, 10 minutes), separated by sodium dodecyl sulfate–polyacrylamide gel electrophoresis, and transferred to PVDF membranes. After blocking with 5% BSA at 37 °C for 30 minutes, the membranes were incubated overnight at 4 °C with primary antibodies against ENTPD1, CYP24A1, TDO2, and β-actin (anti-ENTPD1, GB-111582-50, 1:2000, Servicebio; anti-CYP24A1, GB-113873-50, 1:3000, Servicebio; anti-TDO2, GB-113244-50, 1:1000, Servicebio; anti–β-actin, 66009-1-Ig, 1:25,000, Proteintech). After washing the membranes in TBST, they were incubated with secondary antibodies (HRP-conjugated goat anti-rabbit IgG, GB23303, 1:3000, Proteintech; HRP-conjugated goat anti-rat IgG, GB23301, 1:5000, Proteintech) at room temperature for 30 minutes. The bands were detected with enhanced chemiluminescence reagent and visualised on a gel imaging system. All experiments were performed in triplicate. Image analysis was done using ImageJ software (v 1.43). The mRNA expression of the biomarkers was measured in the gingival tissues (stored at −80 °C) of the PD and control group rats by RT-qPCR, following the same method as described above. The gene-specific primers are listed in Supplementary Table 3. Intergroup differences were assessed using GraphPad Prism software (v 10.0) with a *t* test (*P* < .05).

### Statistical analysis

R language (v 4.3.3) was used for bioinformatics analyses, and group differences were assessed using the Wilcoxon rank-sum test and a *t* test with a significance threshold of *P* < .05.

## Results

### Specific functions of candidate mitochondrial metabolism-related genes

**Fig. 1:**
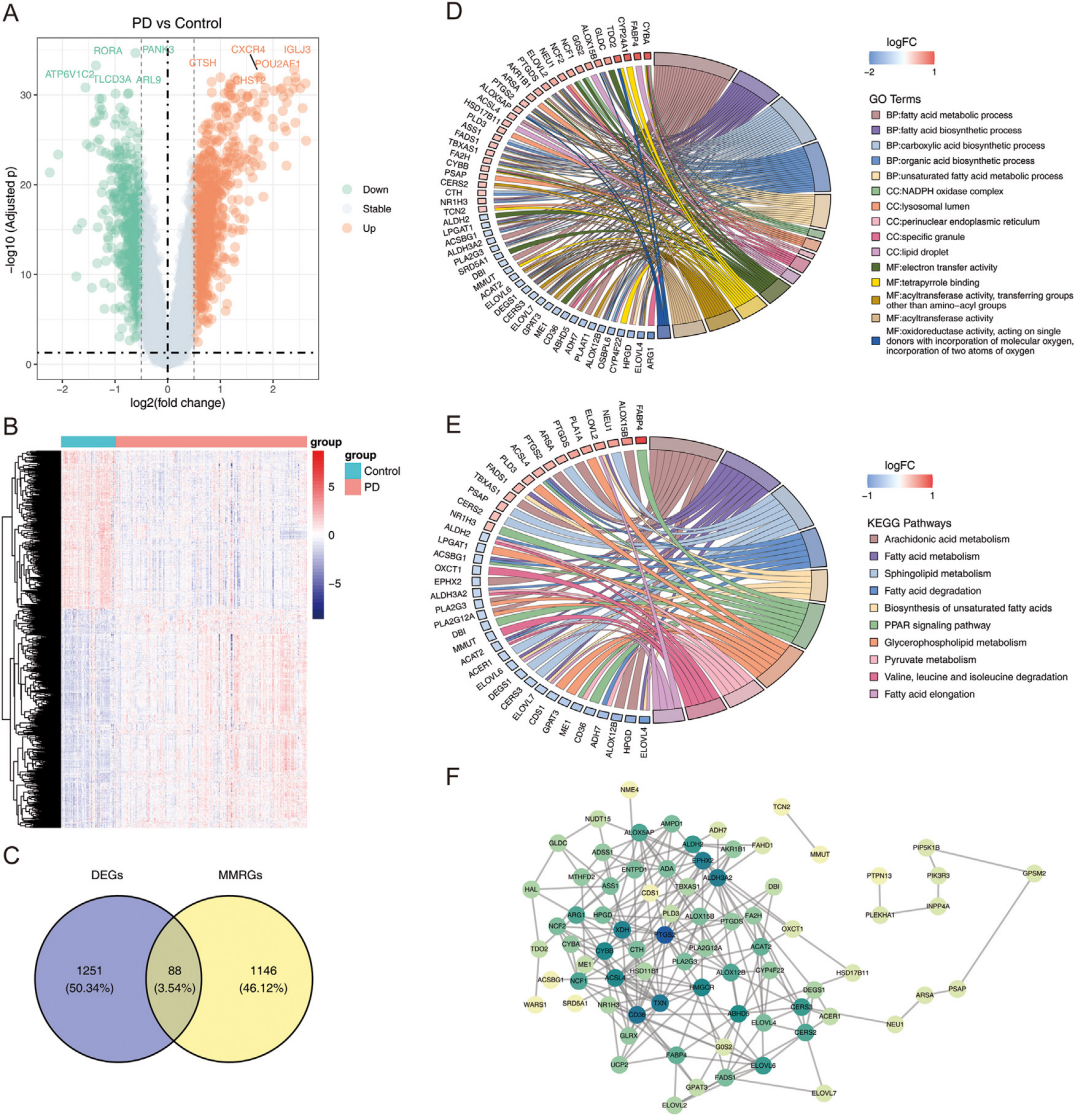
Identification and functional characterisation of mitochondrial metabolism-related candidate genes in periodontitis. (A) Volcano plot of differentially expressed genes (DEGs) in GSE16134. (B) Heatmap of DEGs. (C) Venn diagram showing the intersection of DEGs and mitochondrial metabolism-related genes (MMRGs). (D) Gene Ontology enrichment analysis of candidate genes. (E) Kyoto Encyclopedia of Genes and Genomes enrichment analysis of candidate genes. (F) Protein-protein interaction network of candidate genes.

Using the GSE16134 dataset, 1339 DEGs were identified in the PD group (777 upregulated, 562 downregulated; adjusted *P* < .05) (Figure 1A, B). The intersection of the DEGs and MMRGs yielded 88 candidate genes associated with mitochondrial metabolism in PD (Figure 1C). These candidate genes were significantly enriched in GO terms, including “fatty acid metabolic process” and “NADPH oxidase complex” (*P* < .05) (Figure 1D). They were also associated with the KEGG pathways “arachidonic acid metabolism” and “fatty acid metabolism” (*P* < .05) (Figure 1E). These results clarified the multifaceted role of mitochondrial metabolism in PD progression. In addition, the protein-protein interaction network revealed various interaction pairs (eg, PTGS2-XDH and CD36-TXN), illustrating the complex interplay among candidate genes at the protein level (Figure 1F).

### Recognition of mitochondrial metabolism-related biomarkers with superior diagnostic value

**Fig. 2:**
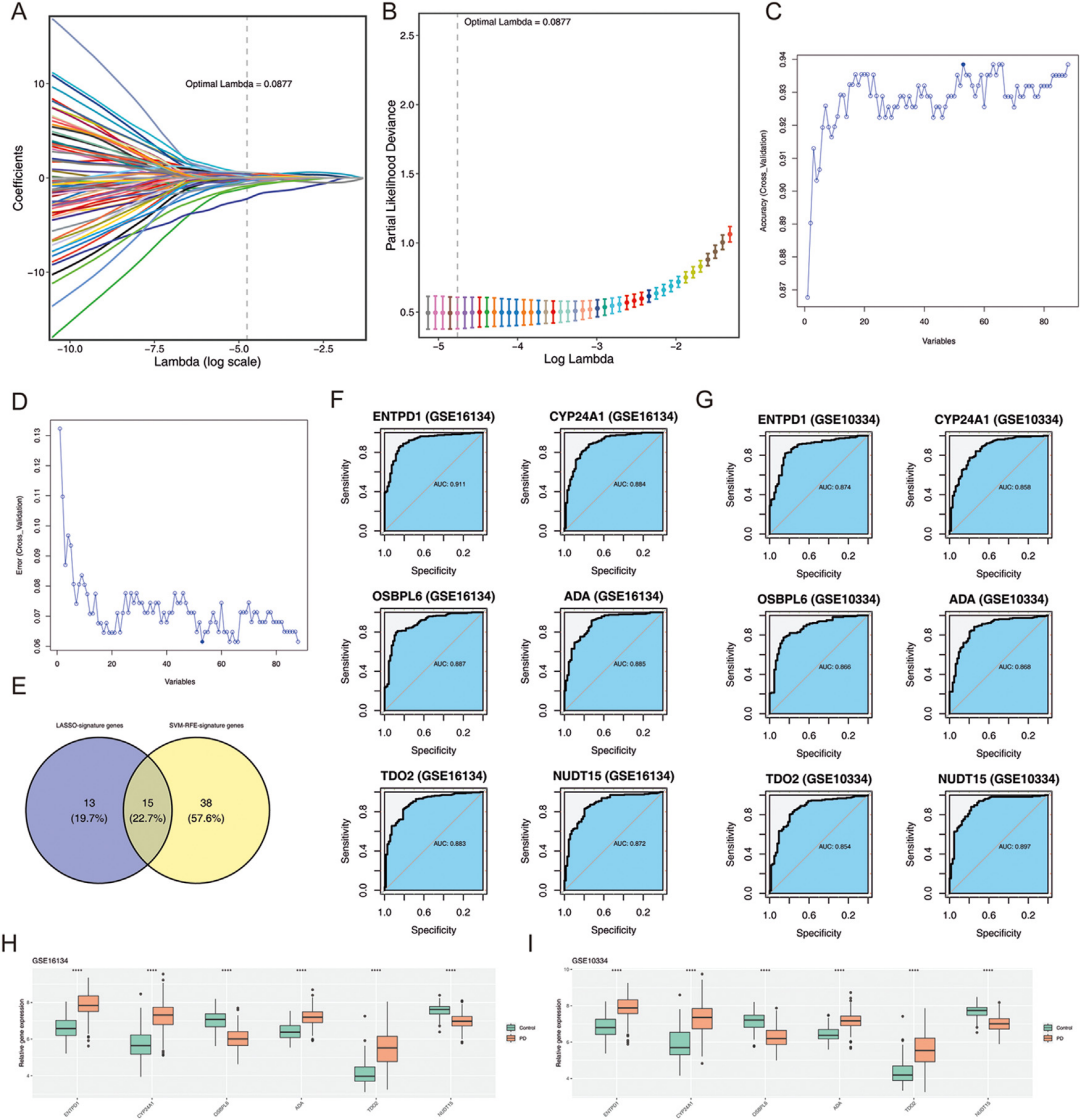
Machine learning–based identification and external validation of mitochondrial metabolism-related biomarkers in periodontitis. (A, B) Least absolute shrinkage and selection operator (LASSO) regression analysis. (C, D) Support vector machine–recursive feature elimination (SVM-RFE) analysis. (E) Venn diagram of overlapping signature genes identified by LASSO and SVM-RFE. (F, G) Receiver operating characteristic curves of candidate biomarkers in GSE16134 and GSE10334. (H, I) Expression validation of candidate biomarkers in GSE16134 and GSE10334. ns, nonsignificant. *P < .05, **P < .01, ***P < .001, ****P < .0001.

Using machine learning algorithms on the GSE16134 dataset, 28 LASSO-signature genes (optimal λ = 0.0877) and 53 SVM-RFE signature genes were identified (Figure 2A-D). From the intersection, 15 signature genes were identified (Figure 2E). ROC curves for 6 signature genes in GSE16134 and GSE10334 exhibited AUC values >0.8, indicating strong discriminatory power for PD (Figure 2F, G). Therefore, these 6 genes were selected as candidate biomarkers. The candidate biomarkers, ectonucleoside triphosphate diphosphohydrolase 1 (ENTPD1), cytochrome P450 family 24 subfamily A member 1 (CYP24A1), adenosine deaminase (ADA), and tryptophan 2,3-dioxygenase (TDO2) were consistently upregulated in PD among both datasets. In contrast, oxysterol binding protein like 6 (OSBPL6) and nudix hydrolase 15 (NUDT15) exhibited decreased expression (Figure 2H, I). Based on these results, ENTPD1, CYP24A1, OSBPL6, ADA, TDO2, and NUDT15 were selected as candidate biomarkers.

**Fig. 3:**
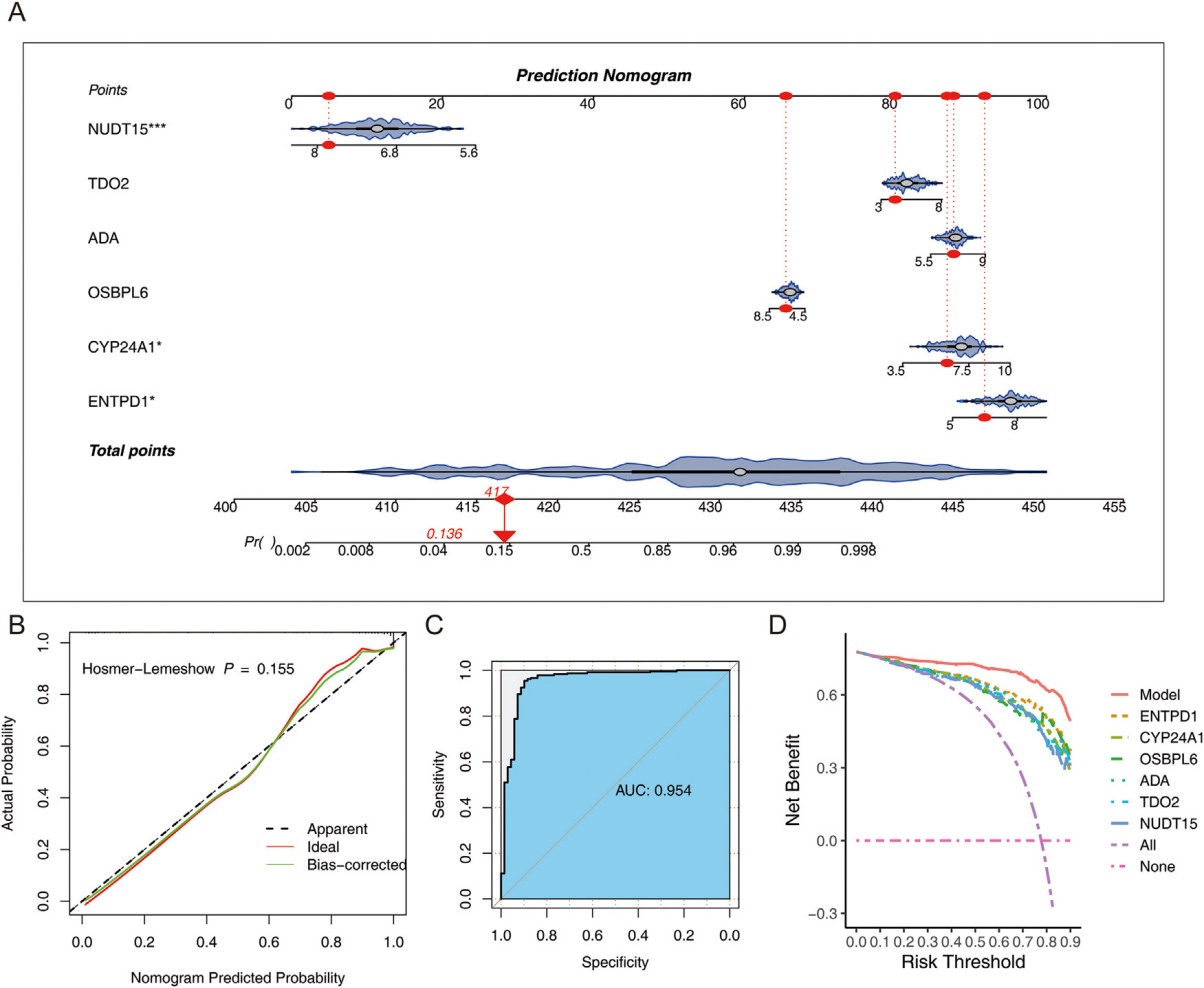
Construction and evaluation of the diagnostic nomogram for periodontitis. (A) Nomogram based on 6 mitochondrial metabolism-related biomarkers. (B) Calibration curve of the nomogram. (C) Receiver operating characteristic curve evaluating diagnostic performance. (D) Decision curve analysis assessing clinical utility.

In the GSE16134 dataset, a diagnostic nomogram model for PD was established based on 6 mitochondria‑related metabolic biomarkers (Figure 3A). A higher total score of the model indicated a greater risk of disease. Internal evaluation of the training set revealed that the calibration curve had a slope close to 1 (*P* > .05) and an AUC value of 0.954 (Figure 3B, C). The DCA decision curve indicated that the model provided a certain clinical net benefit (Figure 3D). Using GSE10334 as an independent external validation cohort, the expression values for the biomarkers in each sample were substituted into the above fixed model for prediction. The calibration curve still showed good agreement (Supplementary Figure 1A), and the ROC analysis yielded an AUC value of 0.933 (Supplementary Figure 1B). The DCA curve also indicated that the net benefit of the model was superior to that of the extreme strategy curves (Supplementary Figure 1C). The external validation AUC was similar to that obtained for the training set, suggesting that the model had a certain generalisation ability.

### Significant correlations and crucial functional pathways of biomarkers

**Fig. 4:**
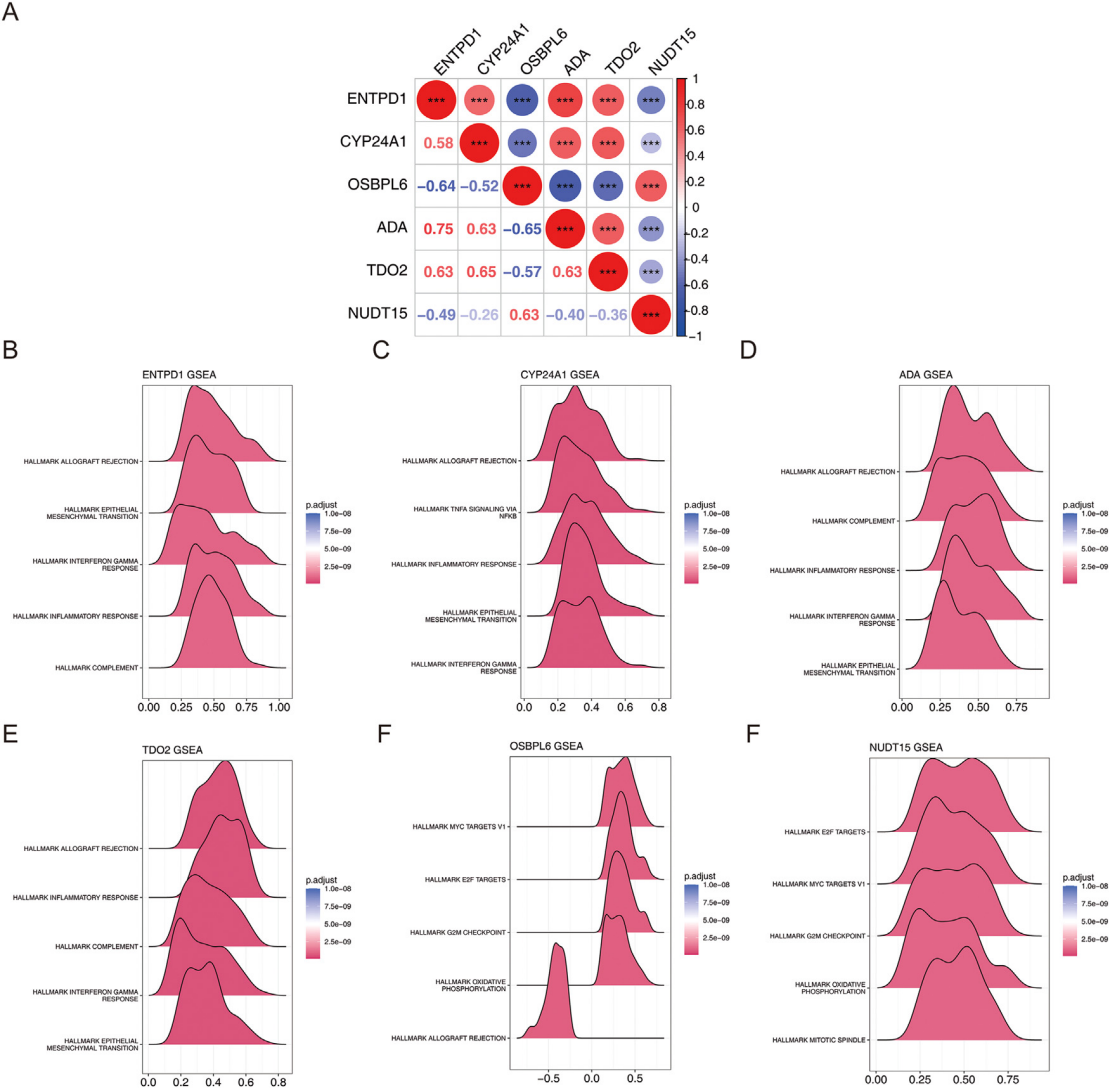
Correlation analysis and gene set enrichment analysis (GSEA) of mitochondrial metabolism-related biomarkers. (A) Correlation heatmap among the identified biomarkers. (B-G) GSEA plots for ENTPD1, CYP24A1, ADA, TDO2, OSBPL6, and NUDT15, respectively.

Remarkably, all mitochondrial metabolism-related biomarkers showed significant correlations with one another (*P* < .001). The strongest positive correlations were between ADA and ENTPD1 (cor = 0.75) and negative between ADA and OSBPL6 (cor = −0.65) (Figure 4A). These results suggest that the biomarkers play important roles in PD progression through highly coordinated regulatory networks.

The biological pathways associated with the biomarkers were determined by GSEA using the GSE16134 dataset. ENTPD1, CYP24A1, ADA, and TDO2 were associated with “allograft rejection,” “epithelial-mesenchymal transition,” and “inflammatory response,” whereas OSBPL6 and NUDT15 were associated with “MYC targets V1,” “E2F targets,” and “oxidative phosphorylation” (adjusted *P* < .*05*) (Figure 4B-G). This suggests that mitochondrial metabolism influences the progression of PD by modulating these pathways.

### Altered immune microenvironment profiles associated with the biomarkers

**Fig. 5:**
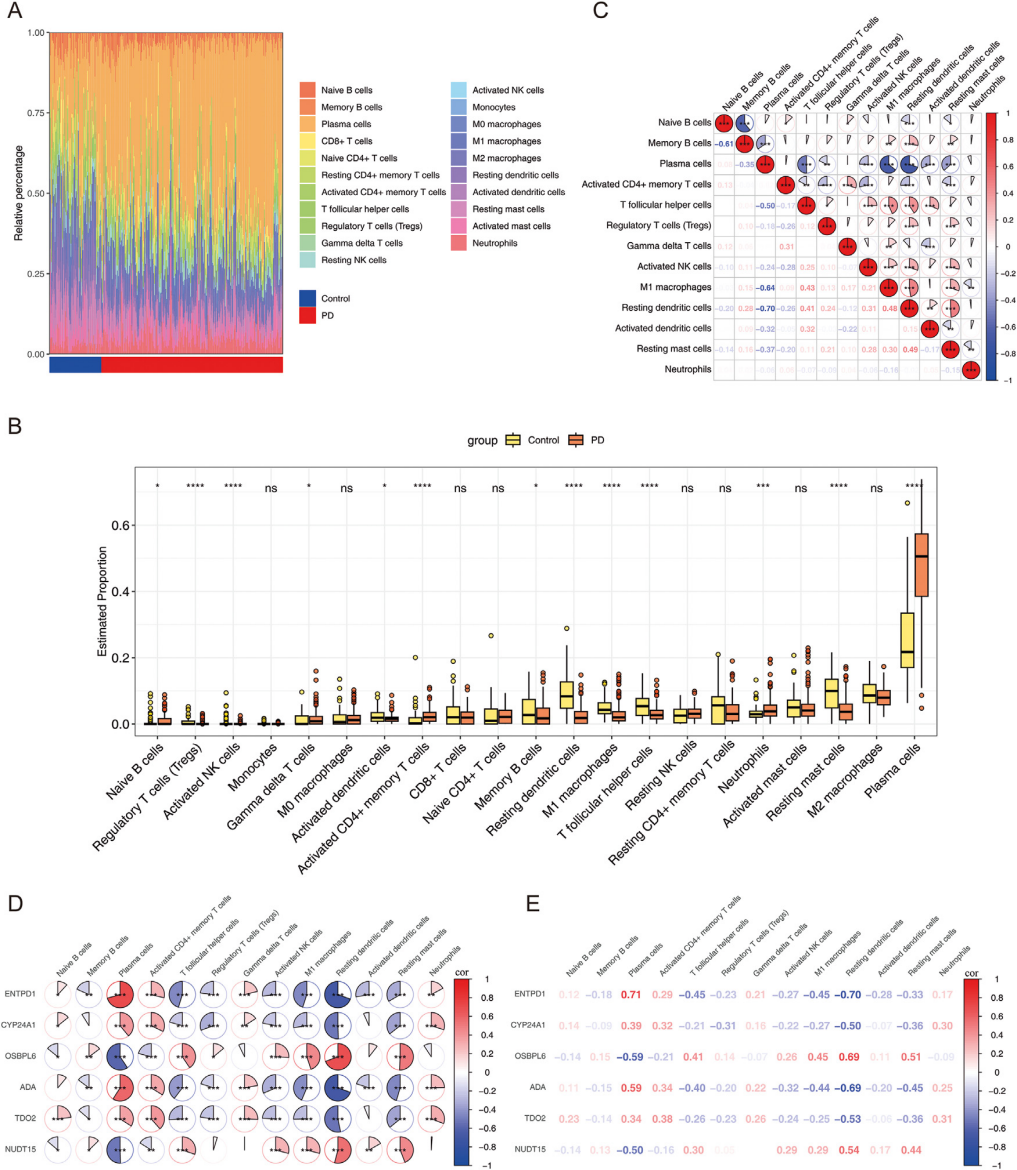
Immune infiltration characteristics associated with mitochondrial metabolism-related biomarkers in periodontitis. (A) Immune cell infiltration landscape in GSE16134. (B) Differentially infiltrating immune cell types between periodontitis and control samples. (C) Correlation analysis among differential immune cell types. (D) Correlation analysis between biomarkers and differential immune cell types. ns, insignificant. *P < .05, **P < .01, ***P < .001, ****P < .0001.

After excluding cell types with zero infiltration across all samples in GSE16134, differential immune microenvironment profiles between the PD and control groups were delineated. Plasma cells exhibited relatively high infiltration (Figure 5A). Thirteen differential immune infiltrating cell types were identified, including plasma cells, resting mast cells, and resting dendritic cells (*P* < .05) (Figure 5B). Significant correlations were observed among the specific cell types (Figure 5C). Resting mast cells and resting dendritic cells exhibited the strongest positive correlation (cor = 0.49, *P* < .001), whereas plasma cells and resting dendritic cells showed the strongest negative correlation (cor = −0.70, *P* < .001). Mitochondrial metabolism-related biomarkers were significantly correlated with most differential immune cell types (Figure 5D). Plasma cells, resting mast cells, and resting dendritic cells correlated with most biomarkers. The strongest positive correlation was observed between plasma cells and ENTPD1 (cor = 0.71), and the strongest negative correlation was between resting dendritic cells and ENTPD1 (cor = −0.70) (*P* < .001). These results indicate that there is an association between the immune cell infiltration features estimated by CIBERSORT and the mitochondria-related metabolic biomarkers. These immune cell types and their relationship with PD progression warrant further study.

### Potent binding of biomarkers with specific small-molecule compounds

**Fig. 6:**
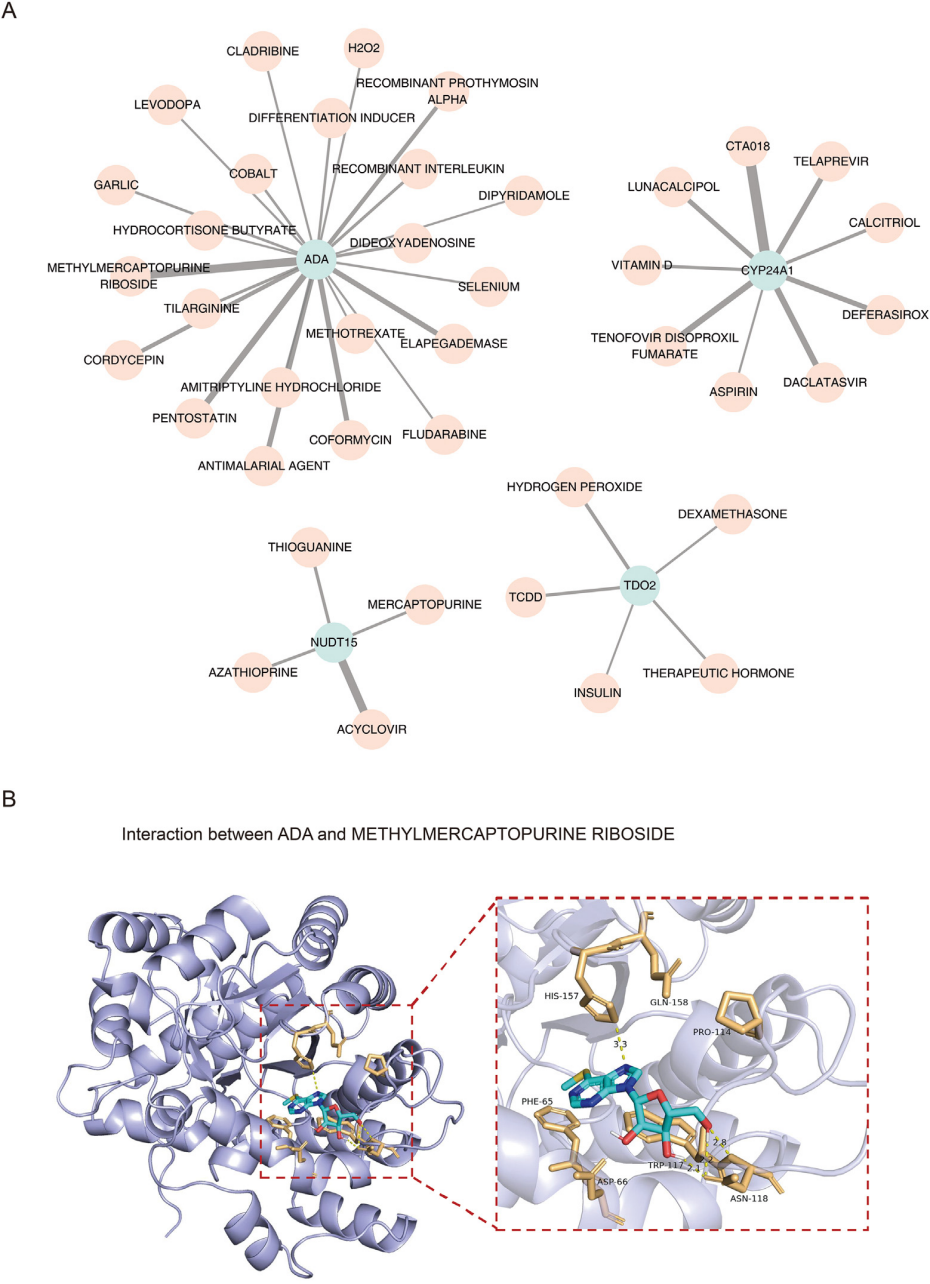
Prediction of small-molecule compounds and molecular docking of mitochondrial metabolism-related biomarkers. (A) Drug-gene interaction network of candidate biomarkers and predicted compounds. (B) Molecular docking models showing the optimal binding conformations of key biomarker-compound pairs.

Among the mitochondria-related metabolic biomarkers, ADA, CYP24A1, NUDT15, and TDO2 were predicted to interact with various small-molecule compounds. The compounds with the highest interaction scores were selected for molecular docking as follows: ADA with mercaptopurine ribonucleoside, CYP24A1 with CTA018, NUDT15 with acyclovir, and TDO2 with hydrogen peroxide (Figure 6A). The optimal binding energies for these biomarker-compound complexes were −5.9, −8.1, −6.8, and −2.7 kcal/mol, respectively (Figure 6B). Based on the preset threshold (binding energy <−5.0 kcal/mol), the interactions between ADA and mercaptopurine ribonucleoside, CYP24A1 and CTA018, and NUDT15 and acyclovir were considered relatively stable. In contrast, the binding energy of the TDO2–hydrogen peroxide complex (−2.7 kcal/mol) did not meet the criterion for strong binding. Although hydrogen peroxide is a well‑known broad‑spectrum antimicrobial agent in periodontal therapy and was the most suitable binding candidate for TDO2 among the screened compounds, the binding energy fell below the threshold, which suggests that the direct interaction between the two is weak. These results provided valuable insight for developing new therapeutic strategies for PD. The 3 stable interactions provide a foundation for targeted drug exploration. Molecular docking predictions are computational results and not equivalent to experimental validation; therefore, the actual binding ability and therapeutic relevance of these compounds require further validation in vitro and in vivo.

### Various regulatory elements targeting biomarkers

**Fig. 7:**
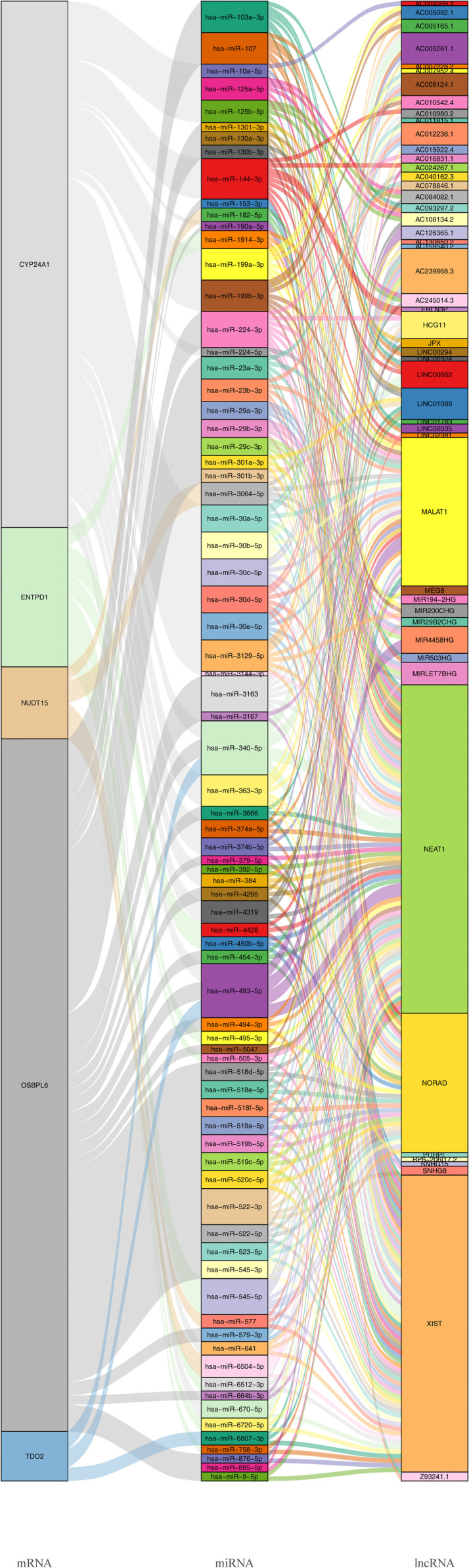
Long noncoding RNA (lncRNA), microRNA (miRNA), and messenger RNA (mRNA) regulatory network of mitochondrial metabolism-related biomarkers in periodontitis.

Various upstream regulatory elements of mitochondrial metabolism-related biomarkers were predicted. The constructed lncRNA-miRNA-mRNA regulatory network consisted of 77 miRNAs, 50 lncRNAs, and 5 biomarkers. (Figure 7). This network encompassed several lncRNA-miRNA-mRNA regulatory relationships, such as AC084082.1–hsa-miR-125a-5p–CYP24A1, MALAT1–hsa-miR-1914-3p–ENTPD1, MALAT1–hsa-miR-3064-5p–NUDT15, NORAD–hsa-miR-518d-5p–OSBPL6, and XIST–hsa-miR-6807-3p–TDO2. Elucidating the regulatory elements of the biomarkers is important for understanding the mechanisms through which mitochondrial metabolism influences PD progression.

### Identification of T cells as a key cell type

**Fig. 8:**
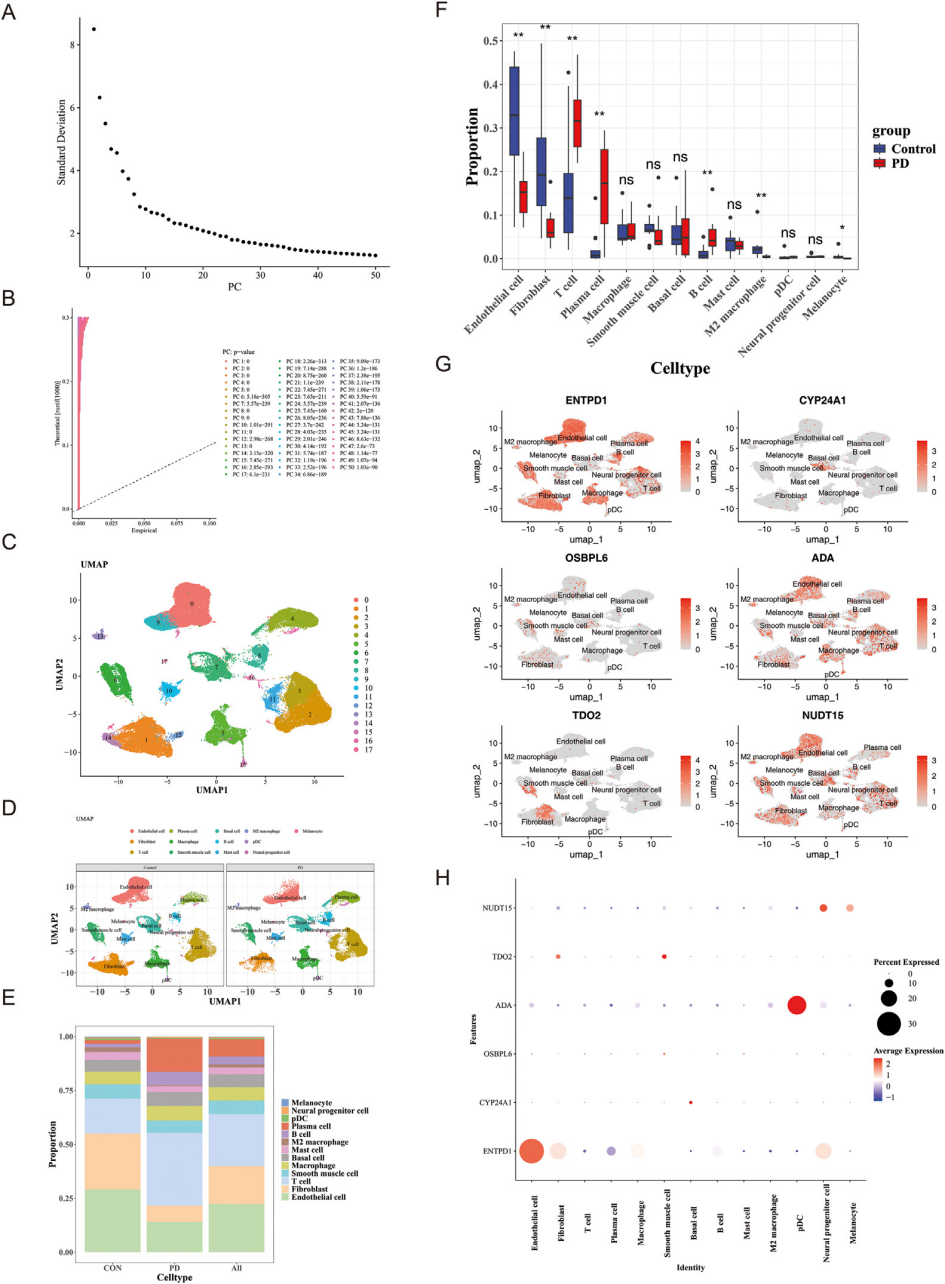
Single-cell transcriptomic landscape and identification of T cells as a key cell type in periodontitis. (A, B) Selection of principal components for downstream analysis. (C) Uniform manifold approximation and projection clustering of single cells. (D) Annotation of major cell types. (E) Cell-type proportion distribution in control and periodontitis samples. (F) Differential cell-type proportions between groups. ns, insignificant. *P < .05, **P < .01. (G, H) Distribution and expression of mitochondrial metabolism-related biomarkers across cell types.

Potential cellular mechanisms underlying PD progression were explored at the single-cell level using the GSE164241 dataset. After Quality Control, 77,304 cells and 23,816 genes were retained (Supplementary Figure 2A, B). The gene variations are shown in Supplementary Figure 1C, highlighting the top 10 Highly Variable Genes. PCA revealed minimal batch effects, with cells from different samples largely intermixed (Supplementary Figure 2D). The top 30 PCs were selected for uniform manifold approximation and projection dimension reduction based on an elbow plot and a PCA permutation test (*P* < .0001) (Figure 8A, B). Clustering identified 18 distinct cell clusters (Figure 8C). Based on the marker genes (Supplementary Table 4), these cell clusters were annotated to 13 major cell types, including endothelial cells, plasma cells, basal cells, M2 macrophages, melanocytes, fibroblasts, macrophages, B cells, plasmacytoid dendritic cells, T cells, smooth muscle cells, mast cells, and neural progenitor cells (Figure 8D). The marker genes were specifically expressed in their corresponding cell clusters and types (Supplementary Figure 3A, B). The proportions of the different cell types are shown in Figure 8E. T cells were the most abundant cell type in the PD group. Notably, 7 cell types (endothelial cells, plasma cells, M2 macrophages, melanocytes, fibroblasts, B cells, and T cells) exhibited significant differences in abundance between the PD and control groups (*P* < .05) (Figure 8F). Because of their high proportion in the PD group and significant intergroup difference, T cells were identified as key cells. Figure 8G and H illustrate the distribution and expression of mitochondrial metabolism-related biomarkers among the distinct cell types. ENTPD1, ADA, and NUDT15 exhibited a high distribution and expression in T cells. These results indicate that many cell types, particularly T cells, have a strong correlation with PD progression.

### Functional exploration and altered cell-cell communication patterns

**Fig. 9:**
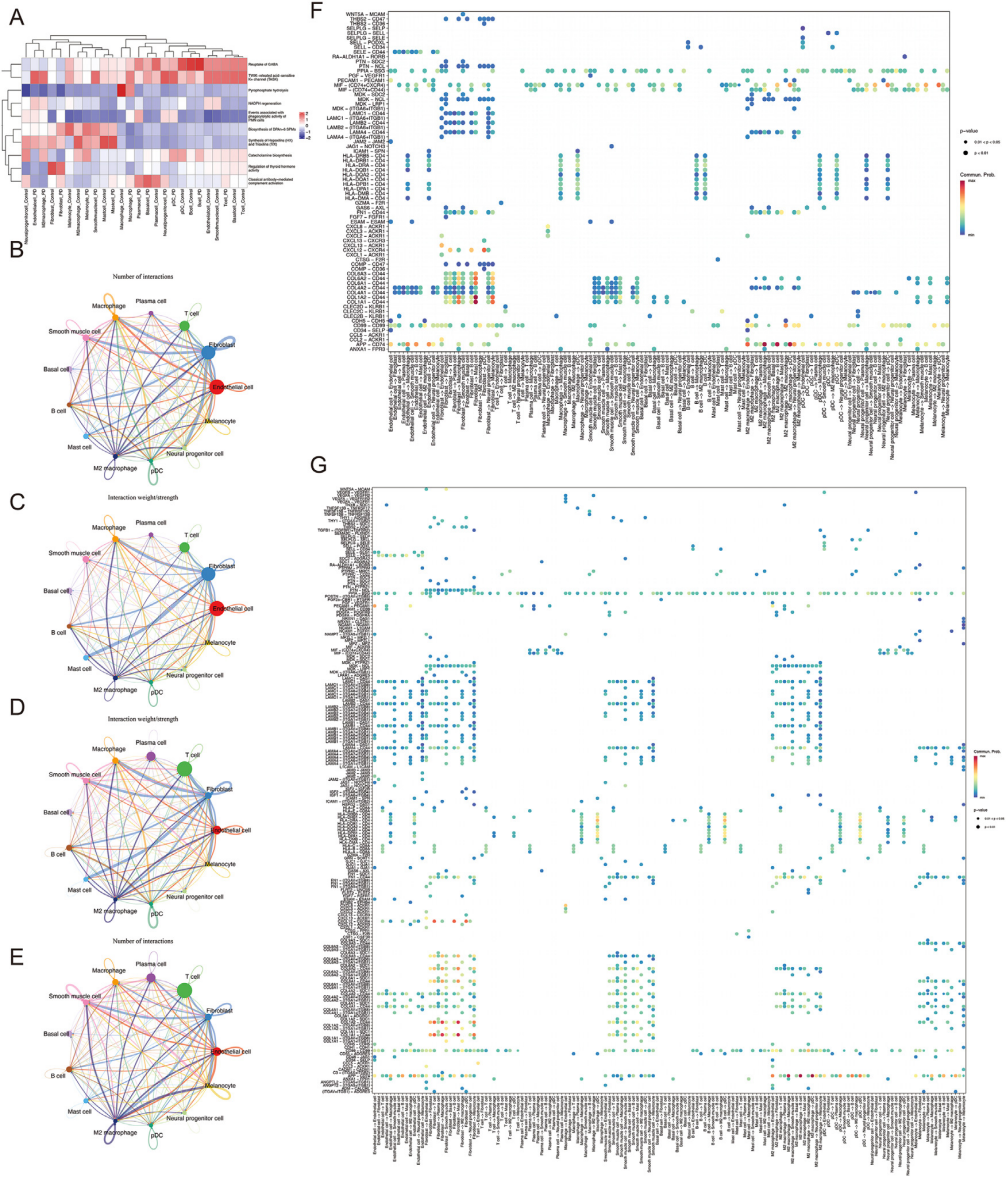
Functional exploration and cell-cell communication analysis of different cell types in periodontitis. (A) Differential pathway activities across cell types. (B-E) Global intercellular communication networks in control and periodontitis groups, shown by interaction number and interaction strength. (F, G) T-cell–centred communication patterns in control and periodontitis groups.

Within GSE164241, the functional roles of all cell types in the PD and control groups were examined (Figure 9A). The key cell type (T cells) was involved in various functional pathways, including “Reuptake of GABA” and “TWIK-related acid-sensitive K^+^ channel (TASK).” Elucidating these cell-type–specific functions enhances our understanding of the cellular processes potentially involved in PD progression.

The analysis of cell-cell communications in the PD and control groups within the GSE164241 dataset revealed extensive intercellular interactions among the cell types (Figure 9B-E). In the control and PD groups, interactions involving fibroblasts showed greater numbers and stronger intensities. T cells were given particular attention as the key cell type. In both groups, T cells interacted with all other cell types, with the interactions between T cells and fibroblasts being the most numerous and strongest. Compared with the control group, the PD group exhibited a marked reduction in the number of interactions between T cells and fibroblasts, whereas the interaction intensities between T cells and fibroblasts and between T cells and smooth muscle cellss were significantly enhanced. Numerous L-R pairs were identified as key mediators of intercellular communication (*P* < .05) (Figure 9F, G). Compared with the control group, the PD group involved a greater number of L-R pairs, indicating more complex and diverse cell-cell communication. Of these, the COL1A2-CD44 pair showed the highest communication probability in the control group and involved interactions between fibroblasts and mast cells. The APP-CD74 pair had the highest communication probability in the PD group and involved interactions between M2 macrophages and plasmacytoid dendritic cells. Taken together, these results indicate that the PD group exhibited communication patterns that differed significantly from those in the control group. In particular, intercellular communications involving T cells were markedly altered and likely contribute to PD progression.

### Dynamic alterations in biomarker expression during T-cell differentiation

**Fig. 10:**
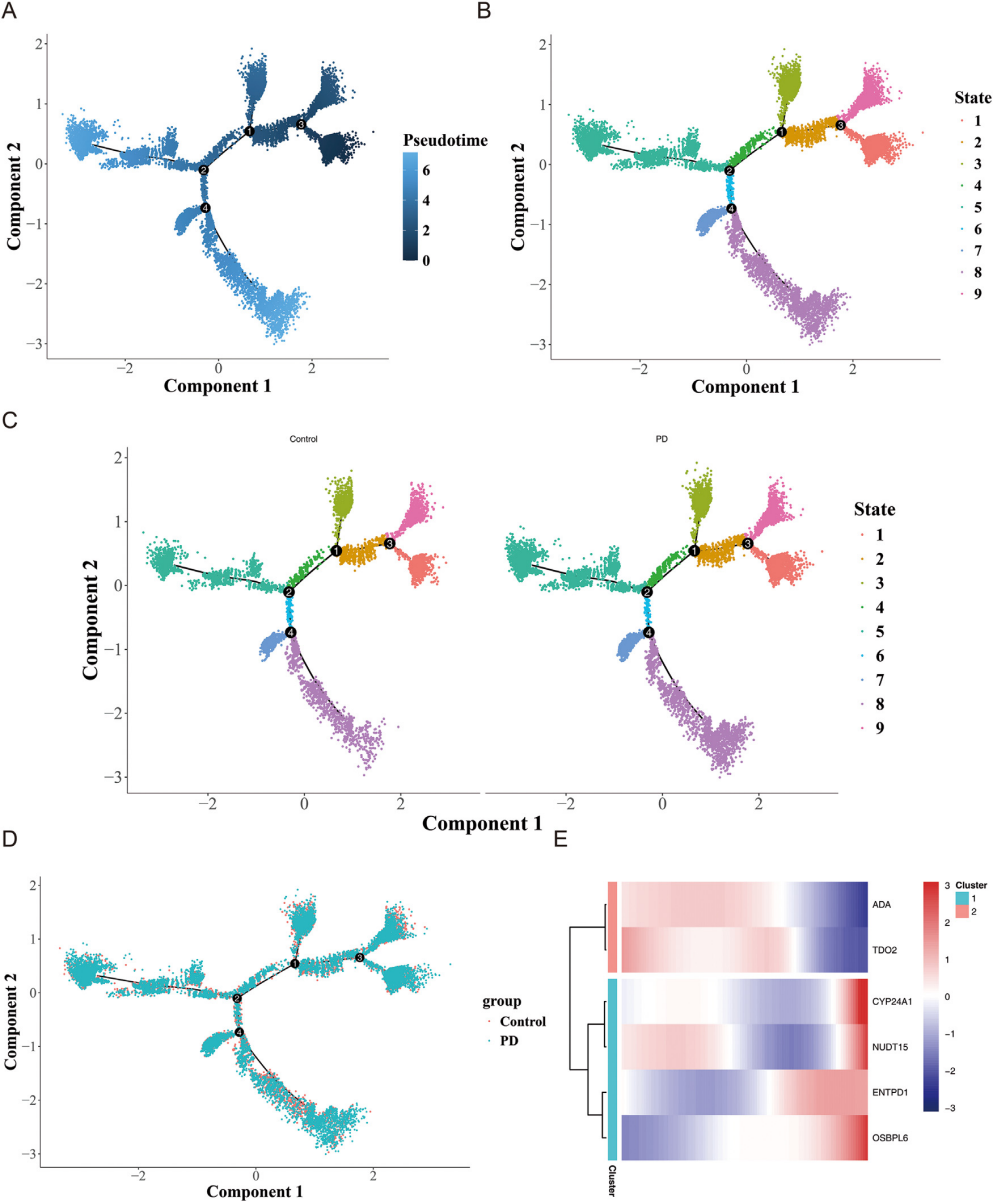
Pseudotime trajectory analysis of T-cell differentiation in periodontitis. (A) Pseudotime trajectory of T cells. (B, C) T-cell differentiation states. (D) Distribution of control and periodontitis T cells along pseudotime. (E) Dynamic expression patterns of mitochondrial metabolism-related biomarkers during T-cell differentiation.

Pseudotime analysis of T cells was performed using the Monocle 2 algorithm to examine the dynamic trends of their transcriptomes. The transition from dark to light colours indicated the direction of the pseudotime progression (Figure 10A). It should be noted that pseudotime analysis is an unsupervised computational ordering method, and the presented order reflects gradual changes in transcriptional similarity rather than a definitive biological differentiation trajectory. The results indicated that T cells were divided into 9 distinct transcriptional states along pseudotime (Figure 10B, C). T cells from the PD and control groups were distributed across these states (Figure 10D). The mitochondria‑related metabolic biomarkers exhibited dynamic expression changes along pseudotime. ENTPD1 first decreased, then increased, and then decreased again, reaching a peak in the middle‑late stage. CYP24A1 first decreased and then increased, peaking in the late stage. OSBPL6 gradually increased, peaking in the late stage. ADA first increased and then decreased, peaking in the early‑middle stage. TDO2 first decreased, then increased, and then decreased again, peaking in the early stage. Finally. NUDT15 first increased, then decreased, and then increased again, peaking in the late stage (Figure 10E). These results suggest that the expression levels of these biomarkers are associated with the transcriptional state transitions of T cells, and their dynamic patterns may be correlated with the progression of PD; however, pseudotime analysis alone could not determine whether these biomarkers were directly involved in regulating T-cell subset differentiation. The causal relationship between them and specific differentiation nodes requires functional validation.

### RT-qPCR validation of the biomarkers

**Fig. 11:**
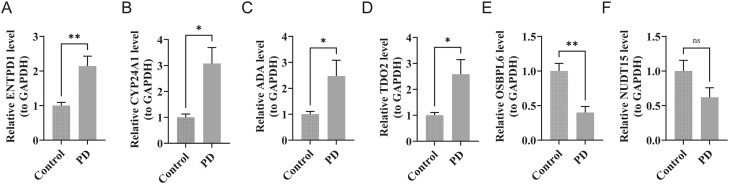
Reverse transcription quantitative polymerase chain reaction validation of mitochondrial metabolism-related biomarkers in clinical samples. (A-F) Expression levels of ENTPD1, CYP24A1, ADA, TDO2, OSBPL6, and NUDT15 in control and periodontitis tissues. ns, nonsignificant. *P < .05, **P < .01, ***P < .001, ****P < .0001.

The expression of ENTPD1, CYP24A1, ADA, and TDO2 was significantly higher in the PD group compared with the normal group, whereas OSBPL6 expression was significantly lower (all *P* < .05) (Figure 11A-E). The expression patterns of these 5 genes were consistent with the results of the preceding dataset analyses. NUDT15 showed only a downward trend in PD, but the difference did not reach statistical significance (Figure 11F). Statistical power analysis revealed that the power values for ENTPD1, CYP24A1, and OSBPL6 were 0.9131, 0.8243, and 0.9609, respectively, all exceeding the commonly used threshold of 0.80. The power for TDO2 and ADA was 0.6820 and 0.5425, respectively, thus remaining at a moderate level. The power for NUDT15 was 0.3659, which was relatively low. In addition, Cohen’s *d* effect sizes for most genes exceeded 1 (Supplementary Table 5). These results suggest that the nonsignificant difference for NUDT15 may be attributable to insufficient statistical power resulting from the small sample size; thus, its potential diagnostic value requires further evaluation in a larger cohort. In contrast, the other 5 genes already exhibited relatively reliable statistical power with the current sample size, and their differential expression further supports their diagnostic value as biomarkers for PD.

### Animal model validation

**Fig. 12:**
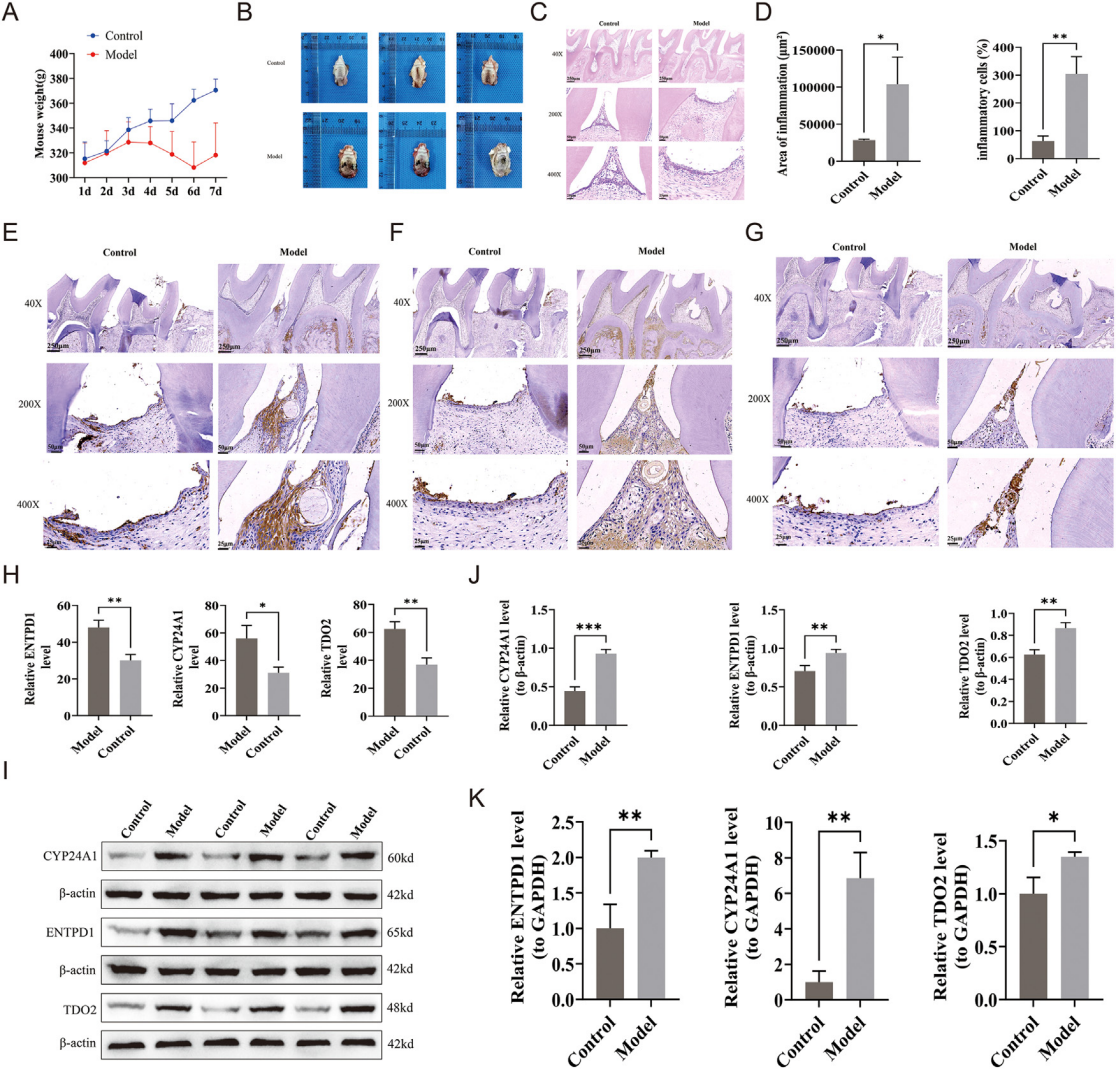
Experimental validation in the rat periodontitis model. (A) Body weight changes during model establishment. (B) Gross morphology of maxillary tissues. (C) Haematoxylin and eosin staining of gingival tissues. (D) Quantification of inflammatory cell percentage and inflammatory area. (E-G) Immunohistochemical staining of ENTPD1, CYP24A1, and TDO2. (H) Quantification of immunohistochemical staining. (I) Western blot results for ENTPD1, CYP24A1, and TDO2. (J) Quantification of Western blot protein expression. (K) Reverse transcription quantitative polymerase chain reaction validation of ENTPD1, CYP24A1, and TDO2 in rat gingival tissues. ns, insignificant. *P < .05, **P < .01, ***P < .001, ****P < .0001.

Six male Sprague-Dawley rats were used to establish a PD model. First, during the housing period, the body weight of the PD group rats increased initially, then decreased, and rose again, with minimal overall change, whereas the body weight of the control group rats gradually increased (Figure 12A). This suggests that PD disrupted the normal physiological growth of the rats. Gross morphologic observation revealed that the maxillae (including periodontal tissues, teeth, and alveolar bone) of the control group rats were structurally intact with uniform pale pink to off-white coloration. No abnormalities (eg, swelling, haemorrhage) were observed around the teeth. In contrast, the periodontal tissues of the PD group rats displayed marked redness and swelling, with local dark red discoloration (indicative of mild haemorrhage), showing significant pathologic changes (Figure 12B).

Haematoxylin and eosin staining revealed that the gingival tissues of the control group rats were structurally intact with regularly arranged cells and only a small number of scattered cells. In contrast, the gingival tissues of the PD group rats exhibited prominent pathologic alterations, including increased cell infiltration and disorganised cell arrangement (Figure 12C). In addition, the percentage of inflammatory cells and the inflammatory area were higher in the PD group compared with those in the control group (*P* < .05), reflecting a relatively pronounced inflammatory response in the gingival tissues of PD rats (Figure 12D).

Because ENTPD1, CYP24A1, and TDO2 exhibited consistent upregulation and the most significant differences in both datasets and clinical samples, we selected these 3 genes as the primary focus for validation in the animal model. IHC, WB, and RT‑qPCR consistently showed that the protein and mRNA expression levels of ENTPD1, CYP24A1, and TDO2 in the gingival tissues of PD rats were higher compared with those in the control group (IHC/WB: *P* < .05; RT‑qPCR: *P* < .01) (Figure 12E-K). These results indicate that the upregulation of these 3 biomarkers is associated with the pathologic changes observed in PD; however, because only 3 animals per group were evaluated, these results should be considered preliminary.

## Discussion

PD is a chronic inflammatory disease of periodontal tissues caused by multifactorial aetiologies.^1^^,^^50^ Mitochondrial metabolism is closely linked to PD pathogenesis and progression.^13^^,^^14^^,^^51^ Through a systematic bioinformatics analysis, we initially screened 6 mitochondrial metabolism-related biomarkers of PD at the transcriptomic level and validated their diagnostic and immunomodulatory value. Subsequently, scRNA‑seq analysis identified T cells as a key cell type in PD progression and revealed the dynamic expression patterns of these biomarkers during T‑cell differentiation as well as their potential roles in cell communication networks. In addition, in vivo validation using a rat model of PD confirmed that the upregulation of ENTPD1, CYP24A1, and TDO2 in periodontal tissues is associated with disease pathogenesis. Based on the associations of the 6 biomarkers identified in the present study with mitochondrial screening and cellular mechanism dissection to animal model validation, these results indicate a role for mitochondrial metabolism in PD pathogenesis, providing a novel theoretical foundation and potential therapeutic targets for diagnosis and treatment.

The association of the 6 biomarkers identified in this study with mitochondrial metabolism was derived from the intersection of the MSigDB database annotations and pathway enrichment analyses, representing a bioinformatics‑driven inferential classification. Therefore, any discussion of their involvement in the regulation of mitochondrial metabolism through specific pathways should be considered mechanistic hypotheses supported by the existing literature, with causality requiring further experimental validation. In this context, the data provide valuable clues regarding the potential roles of these genes in mitochondrial metabolic imbalance during PD. ENTPD1 may participate in the regulation of the mitochondrial stress response through the cAMP signalling pathway.^52^ CYP24A1 may indirectly affect mitochondrial oxidative phosphorylation through vitamin D metabolism.^53^ TDO2 may affect mitochondrial energy production via the tryptophan-NAD⁺ axis.^54^ OSBPL6 and NUDT15 may be associated with the mitochondrial respiratory chain complex and the repair of mitochondrial oxidative damage, respectively.^55^^,^^56^ ADA may be involved in the regulation of mitochondrial function in the inflammatory microenvironment through nucleotide signalling.^57^ GSEA revealed that these biomarkers are coenriched in mitochondria‑related pathways, including “oxidative phosphorylation,” which suggests that mitochondrial metabolic dysfunction may contribute to PD progression. Based on the above hypothesis‑driven framework, the following sections discuss the putative roles of each gene in PD and their interactions with the immune microenvironment.

ENTPD1 encodes CD39, which is an ectonucleotidase that hydrolyses extracellular ATP to adenosine monophosphate (AMP), thereby regulating immune cell activation and chemotaxis by modulating the ATP/adenosine ratio.^58^, ^59^, ^60^, ^61^ In the present study, ENTPD1 upregulation was observed in both bioinformatics datasets, validated by RT-qPCR in clinical samples, and confirmed at the protein and mRNA levels in animal models, thus establishing it as a stable molecular marker of PD.^61^ Immune infiltration analysis revealed that ENTPD1 had the strongest positive correlation with plasma cells (*r* = 0.71) and the strongest negative correlation with resting dendritic cells (*r* = −0.70). Plasma cells are terminally differentiated B lymphocytes that play a dual role in local periodontal inflammation. They contribute to immune clearance but may also exacerbate immune-mediated tissue damage and bone resorption through IL-35 and IL-37 secretion.^62^, ^63^, ^64^ Dendritic cells integrate pathogen and microenvironmental signals, contributing to both immune activation and immune evasion during PD progression, and may act as vectors for disseminating pathogens systemically.^65^, ^66^, ^67^ Considering the established role of mitochondrial metabolism in regulating dendritic cell differentiation,^68^ we hypothesise that ENTPD1 upregulation may promote plasma cell activation and antibody secretion by modulating the extracellular ATP/adenosine ratio, exacerbate local tissue damage, and disrupt the normal differentiation and functional status of dendritic cells. Furthermore, increased CD39 activity can activate the cAMP-mediated mitochondrial stress response,^52^ suggesting that ENTPD1 may synergistically contribute to PD progression through mitochondrial and immunoregulatory pathways.

CYP24A1 encodes vitamin D 24-hydroxylase, which is a key regulator of active vitamin D degradation.^69^ It is involved in calcium and phosphorus metabolism and affects bone mineralisation and skeletal health.^70^^,^^71^ In addition, vitamin D regulates mitochondrial fusion/fission and oxidative phosphorylation.^72^ Based on these findings, we hypothesise that high CYP24A1 expression may cause vitamin D metabolic abnormalities, thereby indirectly reducing mitochondrial metabolic adaptability. Furthermore, it may decrease the inhibitory effect of vitamin D on osteoclast differentiation. When combined with the regulatory role of CYP24A1 during immune cell infiltration.^73^ it may synergistically accelerate alveolar bone resorption. CYP24A1 was highly enriched in the “epithelial-mesenchymal transition” and “inflammatory response” pathways. This suggests its multifaceted role in promoting the destruction of periodontal tissue structure and the maintenance of chronic inflammation, which is consistent with previous reports.^74^ Notably, molecular docking analysis revealed that the small-molecule compound CTA018 binds to CYP24A1 with a binding energy of −8.1 kcal/mol. This was the most stable interaction among all the biomarker-compound pairs tested. These results suggest that targeting CYP24A1 with CTA018 may have value for the development of novel therapeutic strategies for PD.

TDO2 catalyses the oxidative catabolism of tryptophan via the kynurenine pathway,^75^, ^76^, ^77^ thereby influencing NAD⁺ synthesis and mitochondrial energy production and antioxidant capacity.^54^ In the present study, single-cell analysis revealed that TDO2 expression was highest during the early stages of T-cell differentiation, with levels progressively decreasing as differentiation proceeded. TDO2 suppresses effector T-cell activation and promotes Treg generation via the kynurenine pathway.^78^ This functional mechanism is consistent with the known Th17/Treg imbalance that occurs in PD^79^ and the altered T cell communication patterns observed in the PD group in the present study. Nevertheless, pseudotime analysis only reflects gene expression trends along pseudotime and does not establish that changes in TDO2 expression directly cause an imbalance in T-cell subset differentiation. This causal relationship requires further study. Furthermore, RT-qPCR analysis of the clinical samples and experiments in rat periodontal tissues confirmed the significant upregulation of TDO2, which provides in vivo support for the proposed mechanism.

In the present study, ADA was significantly upregulated in PD and showed the strongest positive correlation with ENTPD1 (*r* = 0.75). ENTPD1/CD39 hydrolyses extracellular ATP to generate AMP,^58^, ^59^, ^60^, ^61^ whereas ADA converts adenosine, a metabolite of AMP, into inosine.^80^ Thus, the 2 enzymes may constitute an upstream-downstream relationship along the extracellular ATP-adenosine metabolic axis to regulate PD progression. Importantly, dysregulation of adenosine levels affects mitochondrial function through adenosine receptor signalling.^57^ Therefore, upregulation of ADA may indirectly participate in mitochondrial metabolic dysregulation by attenuating adenosine levels.

OSBPL6 encodes oxysterol-binding protein-related protein 6 (ORP6), which is involved in intracellular cholesterol transport.^55^ Cholesterol metabolic homeostasis is important for the lipid composition of mitochondrial membranes, and impaired cholesterol transport may indirectly disrupt mitochondrial function.^81^ Consequently, OSBPL6 may exacerbate mitochondrial metabolic imbalance through this pathway and contribute to PD progression. NUDT15 is a member of the Nudix hydrolase superfamily that catalyses the hydrolysis of oxidised nucleotides (eg, 8-oxo-dGTP), thus preventing their incorporation into DNA and subsequent base mismatches.^82^ PD is characterised by local excessive ROS production and oxidative stress.^15^ In this microenvironment, NUDT15 downregulation may lead to insufficient clearance of oxidised nucleotides, thus exacerbating the accumulation of oxidative damage to mitochondrial DNA. Of note, the above mechanistic hypotheses are based on other experimental systems reported in the existing literature. The precise functions of ADA, OSBPL6, and NUDT15 in periodontal tissues, as well as their specific effects on mitochondrial metabolism, require validation in cellular and animal models. The results indicated that ENTPD1, CYP24A1, ADA, and TDO2 were primarily enriched in the epithelial-mesenchymal transition (EMT), inflammatory response, and allograft rejection, whereas OSBPL6 and NUDT15 were primarily enriched in MYC targets V1, E2F targets, and oxidative phosphorylation (OXPHOS) pathways. This suggests that different biomarkers contribute to PD progression through distinct pathways. EMT contributes to periodontal tissue destruction by promoting epithelial-to-fibroblast-like transformation,^83^^,^^84^ which is further exacerbated by mitochondrial dysfunction.^85^ This suggests that ENTPD1 and CYP24A indirectly drive EMT through this pathway. Notably, the enhanced fibroblast interaction intensity observed in cell communication analysis provides indirect support for this EMT-driven mechanism at a cellular level.

The enrichment of the inflammatory response pathway is consistent with the central proinflammatory role of the Th17/IL-23–IL-17 axis in PD.^86^ Mitochondria may participate in this process by regulating immune cell function.^87^ Single-cell analysis in the present study also revealed a significant alteration in T-cell communication patterns in the PD group. Combined with the mechanism by which TDO2 affects T-cell differentiation through the kynurenine pathway, this suggests that the biomarkers may amplify Th17-mediated inflammatory signals via mitochondrial metabolic disruption, thereby exacerbating periodontal tissue damage. The enrichment of OSBPL6 and NUDT15 in the OXPHOS pathway, together with their roles in mitochondrial lipid metabolism and oxidative damage repair, indicates that their downregulation may disrupt immune cell function by compromising mitochondrial oxidative phosphorylation efficiency. OXPHOS not only acts as the energetic foundation for macrophage M2 polarisation^88^, ^89^, ^90^, ^91^ but also provides metabolic support for the high-energy demand required by plasma cells to sustain substantial antibody secretion. This aligns closely with the results of our immune infiltration analysis, which identified plasma cells as the most significant differential infiltrating cell type. The enrichment of the MYC targets V1 and E2F suggests that these 2 genes may also be involved in the dysregulation of cell proliferation and apoptosis in PD.

To further examine the translational potential of the biomarkers, we used molecular docking to screen for small-molecule compounds that bind to them. Computational simulation results indicated that acyclovir binds to NUDT15, methylmercaptopurine riboside binds to ADA, and CTA018 binds to CYP24A1 at relatively low binding energies. This suggests favourable binding tendencies for these pairs. The docking analyses were performed using an online blind docking platform, which did not incorporate factors such as protein conformational dynamics or solvation effects; therefore, the results should be considered preliminary. Nevertheless, these computational results provide a meaningful foundation for subsequent studies. Herpesviruses and periodontal pathogens can synergistically exacerbate tissue destruction,^92^ and an anti-infective regimen involving systemic acyclovir administration demonstrated efficacy in severe PD.^93^ Although the binding energy between hydrogen peroxide and TDO2 in the present study was −2.7 kcal/mol, which did not reach the preset threshold, hydrogen peroxide exhibited the highest interaction score with TDO2 among all compounds tested. Hydrogen peroxide is a broad-spectrum antimicrobial agent that is widely used for the local treatment of PD.^94^ The acyclovir-NUDT15 and hydrogen peroxide–TDO2 pairs predicted in the present study intersect precisely with the above known clinical applications at the target level. This suggests that our findings provide a novel potential direction for the molecular targeted therapy of PD; however, compounds such as CTA018 are not clinically validated agents in the field of dentistry. Future studies are needed to verify the direct binding of these compounds to their target proteins using other methods, such as surface plasmon resonance or cellular thermal shift assays in vitro, and to evaluate their functional effects on mitochondrial metabolism and inflammatory responses in PD models. This will provide experimental support for translation towards clinical applications.

Single-cell RNA sequencing identified T cells as the most abundant and differentially distributed cell type in PD, which highlights the potential role of T-cell–mediated adaptive immunity in disease progression. Pseudotime analysis revealed 9 distinct T-cell differentiation states. Mitochondrial metabolism‑related biomarkers exhibited distinct dynamic expression patterns among these differentiation stages. ENTPD1 and ADA exhibited higher expression in the mid‑to‑late stages of differentiation, whereas TDO2 peaked during the early stage and subsequently declined. This suggests an association between the expression of the above biomarkers and the transition of T-cell transcriptional states. Considering the established pathogenic role of Th17/Treg imbalance in PD,^95^, ^96^, ^97^ these dynamic changes appear to temporally overlap with known features of immune dysregulation. Therefore, we hypothesise that the dysregulated dynamic expression of these biomarkers may disrupt mitochondrial metabolic homeostasis, thereby amplifying proinflammatory signals and impairing immunoregulatory capacity at specific differentiation nodes, thus accelerating periodontal tissue destruction. However, future functional studies, such as T Cell Receptor stimulation assays and Treg/Th17 in vitro polarisation experiments, will be necessary to validate the precise regulatory roles of these biomarkers in T-cell differentiation.

Cell communication analysis revealed that in the PD group, the interaction intensity between T cells and fibroblasts was increased, whereas the number of communications was reduced. This pattern suggests more focused signal exchange between the 2 cell types. The APP-CD74 ligand-receptor pair exhibited a relatively high communication probability in the PD group. Because CD74 functions not only in antigen presentation but also as an inflammatory signal receptor,^98^ T cells may affect the activation status of fibroblasts through this axis. These results suggest that the T-cell–fibroblast axis may play a role in tissue remodelling during PD.

RT-qPCR analysis of clinical samples indicated that the expression trends of ENTPD1, CYP24A1, ADA, TDO2, and OSBPL6 were consistent with the bioinformatics predictions; however, these assays were limited by sample size and should only be considered preliminary. The insignificant downward trend of NUDT15 also reflects the effect of the limited sample size. In the PD animal model, the upregulation of ENTPD1, CYP24A1, and TDO2 at the protein and mRNA level provides preliminary in vivo evidence for the involvement of these molecules in PD progression. Taken together, multilevel analyses suggest that the expression of ENTPD1, CYP24A1, and TDO2 may be associated with PD progression, offering a new analytical framework and candidate biomarkers for understanding its pathogenesis and exploring molecular diagnostics. Future studies should examine the feasibility of detecting these markers noninvasively in saliva or gingival crevicular fluid (GCF), which may enhance their clinical translational value. Because of their ease of collection, noninvasiveness, and repeatability, saliva and GCF are considered ideal sources for the diagnosis of periodontal diseases.^99^ Using high‑throughput proteomic techniques, such as matrix-assisted laser desorption/ionization time-of-flight mass spectrometry, novel protein markers that distinguish periodontal health from PD have been recently identified from GCF and saliva.^100^ Notably, ENTPD1, as a cell membrane–localised ectonucleotidase, may theoretically enter the saliva/GCF through tissue exudation.^101^ Nevertheless, the above propositions still face practical challenges, such as body fluid concentrations, matrix interference, and detection sensitivity, and their feasibility requires systematic validation in clinical studies.

The present study has several limitations. First, the sample size in the datasets used for the bioinformatics analysis was limited; therefore, the generalisability of the conclusions requires validation in larger multicentre clinical cohorts. Second, RT-qPCR validation using clinical samples included only 5 cases per group, which limits the statistical robustness of the results. Moreover, the cross-group expression stability of the reference gene GAPDH was not formally validated with algorithms such as geNorm or NormFinder. Although GAPDH is widely used as a reference gene in periodontal tissues, its expression may fluctuate under inflammatory conditions, and this assumed stability constitutes a methodological limitation. Third, the rat PD model used only 3 animals per group. Although this is sufficient for a pilot proof of concept, the small sample size has limited statistical power for *t* tests, and the results are susceptible to individual outlier effects; thus, they must be interpreted with caution. In addition, this study primarily relied on correlational analyses and bioinformatic inferences. It did not directly validate the causal effects of these biomarkers on mitochondrial metabolism or PD‑related cellular functions through functional experiments. Considering the above limitations, future studies should focus on the following directions: (1) evaluating the diagnostic performance of these biomarkers in larger clinical cohorts and identifying the optimal reference gene from candidates such as ACTB, B2M, and RPLP0 using geNorm or NormFinder algorithms to ensure the reliability of RT-qPCR data; (2) exploring the feasibility of detecting them in noninvasive body fluids, such as saliva or GCF; (3) conducting large‑sample preclinical animal studies to obtain more robust statistical inferences; and (4) validating the functional mechanisms of the biomarkers in PD through in vitro cellular experiments and gene knockout models.

## Conclusion

Preliminary observations from a small sample of clinical specimens and animal models revealed the upregulation of ENTPD1, CYP24A1, and TDO2 in PD, which was associated with the pathologic changes. This study provides preliminary bioinformatic evidence for the involvement of mitochondrial metabolism‑related genes in the pathogenesis of PD and provides a foundation for future research; however, the clinical applicability of these findings can only be assessed after validation in larger clinical cohorts and functional experiments.

## Conflict of interest

None disclosed.

## Appendix Supplementary materials

Image, image 1 dummy alt text

Image, image 2 dummy alt text

Image, image 3 dummy alt text

Image, application 4

Image, application 5

Image, application 6

Image, application 7

**Supplementary Fig 1.** Validation of the nomogram in the verification set. (A) Calibration curve. (B) Receiver operating characteristic curve. (C) Decision curve analysis curve. Image, application 8

## Footnotes

Supplementary material associated with this article can be found in the online version at doi:10.1016/j.identj.2026.109682.
