# Supplementary figures and images for: Mitochondrial Metabolic Biomarkers in Periodontitis: Discovery and Clinical Validation
Source: Int Dent J. 2026 Jun 13;76(4):109682. doi: 10.1016/j.identj.2026.109682 (PMC13279168; doi:10.1016/j.identj.2026.109682)

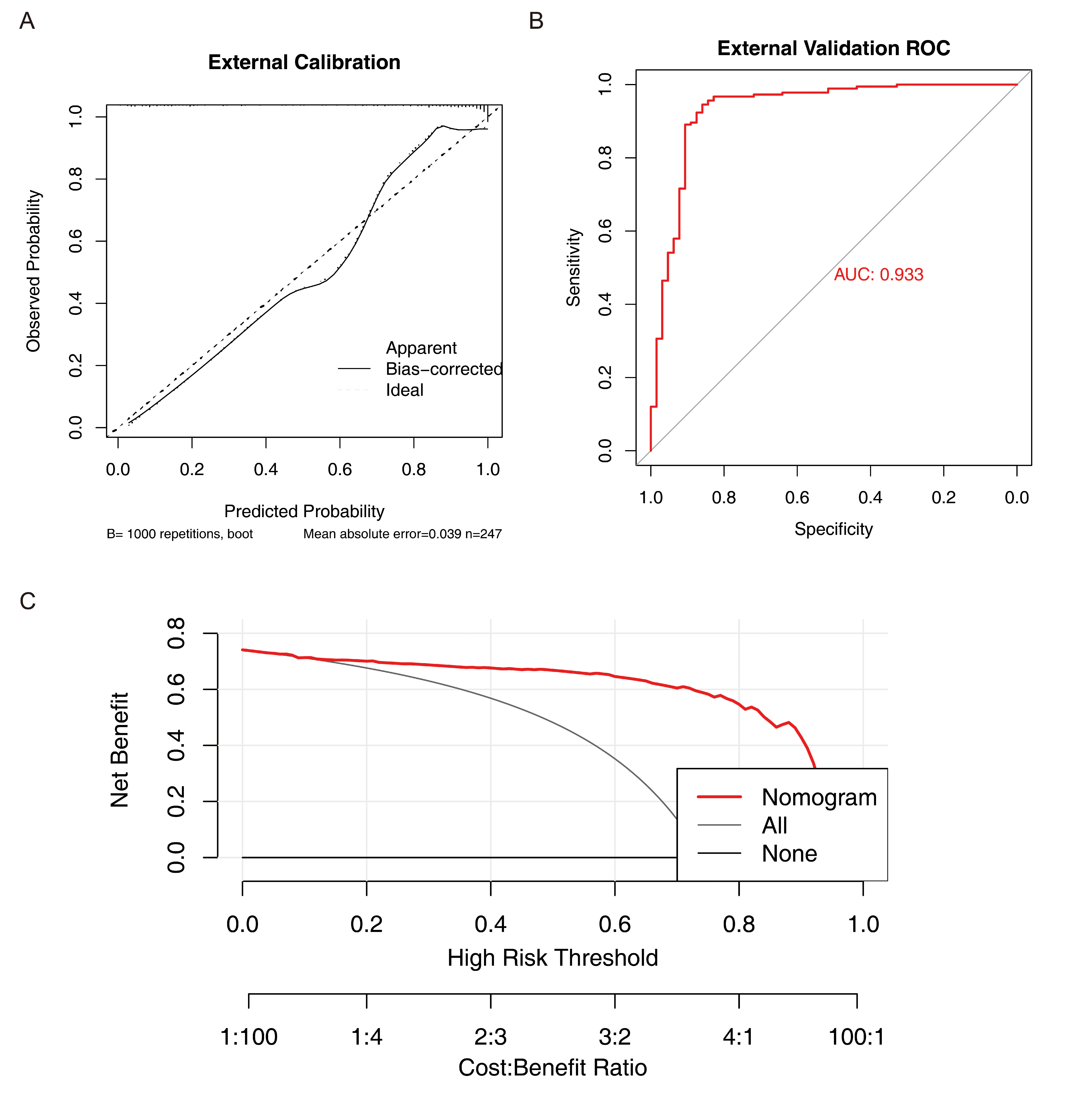

Supplement: Supplementary file 1 [file mmc1.jpg]

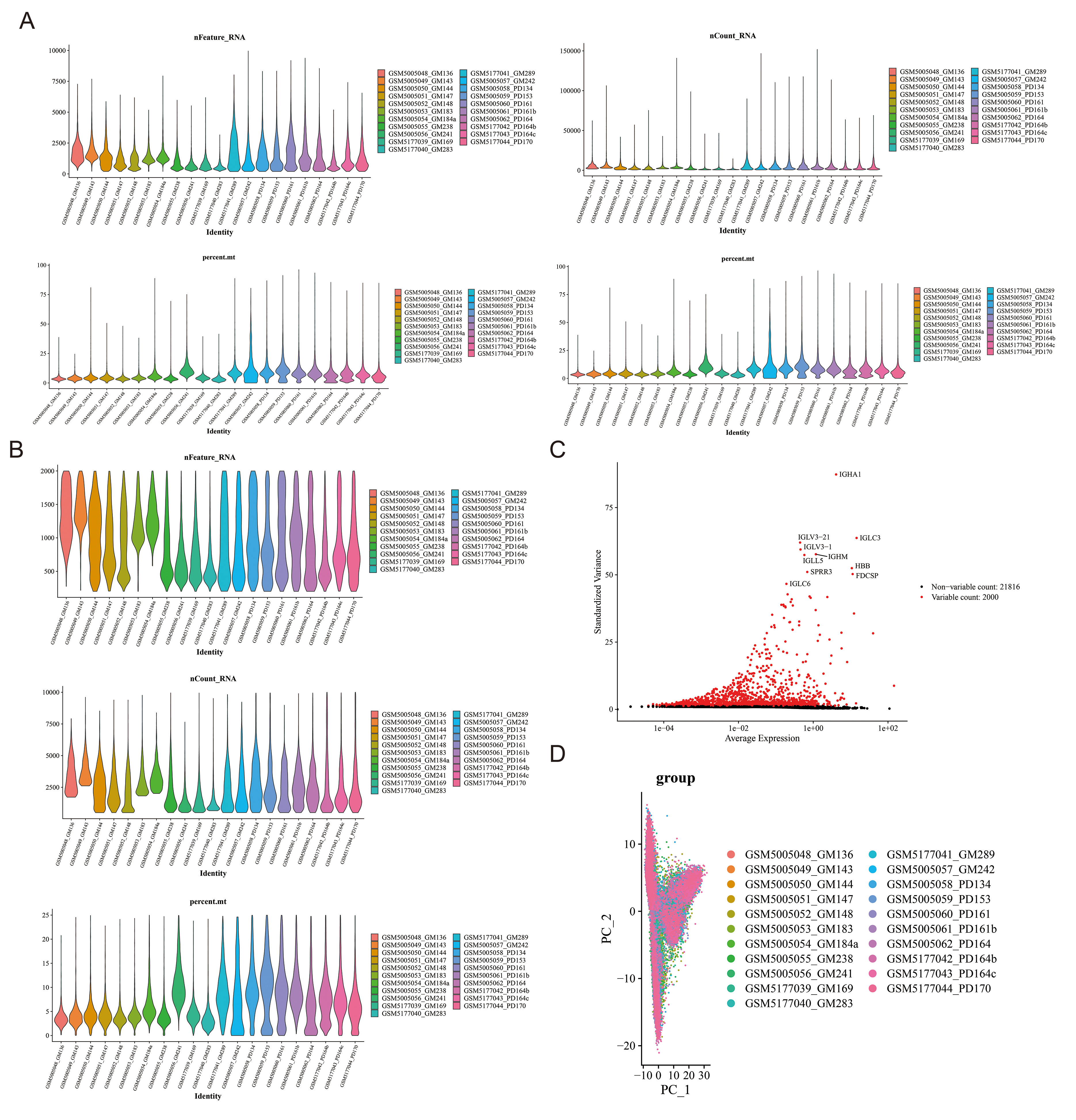

Supplement: Supplementary file 2 [file mmc2.jpg]

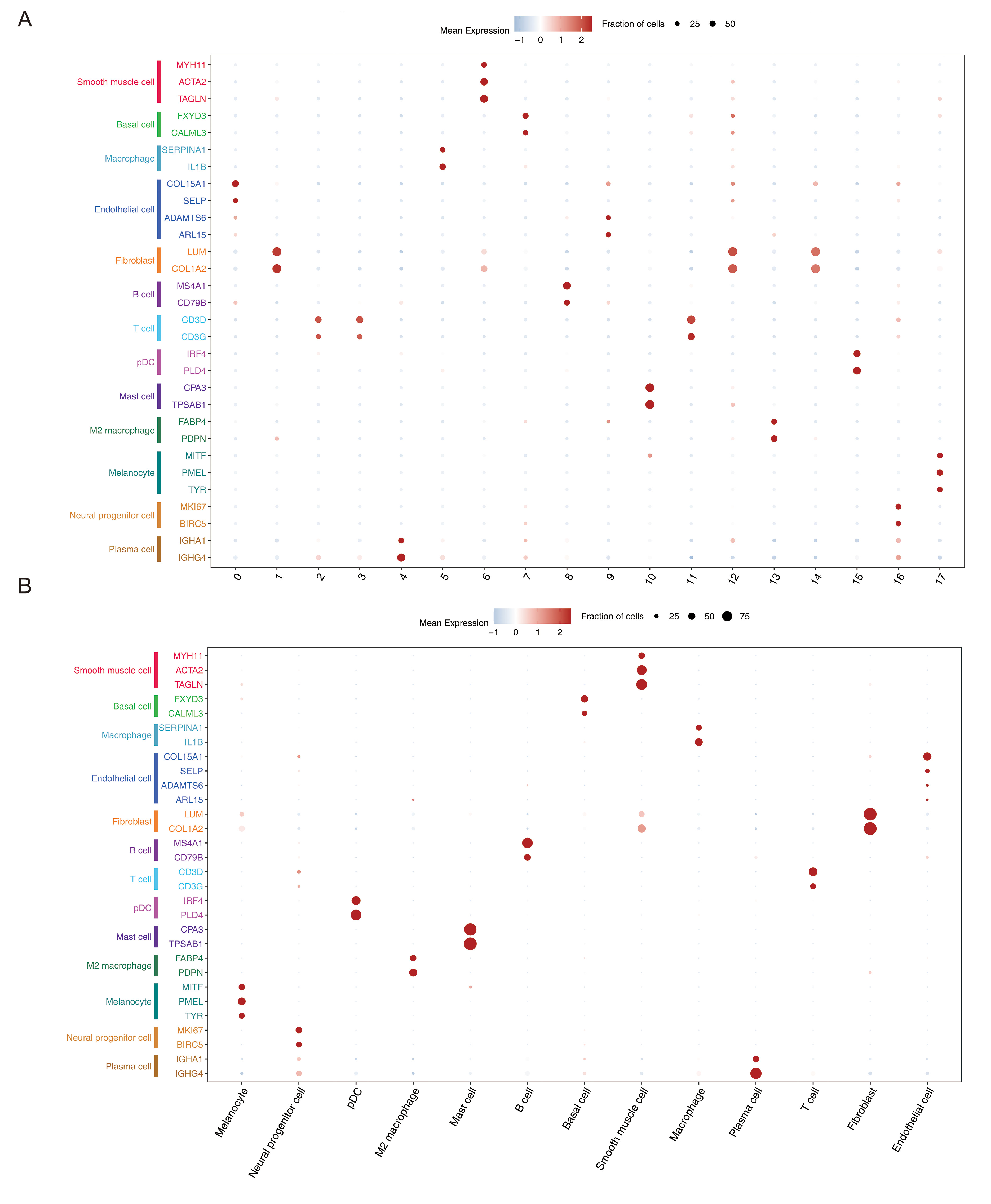

Supplement: Supplementary file 3 [file mmc3.jpg]
