# Supplementary material for: Mitochondrial Metabolic Biomarkers in Periodontitis: Discovery and Clinical Validation
Source: Int Dent J. 2026 Jun 13;76(4):109682. doi: 10.1016/j.identj.2026.109682 (PMC13279168; doi:10.1016/j.identj.2026.109682)
Supplement: Supplementary file 6 [file mmc6.docx]

Supplementary Table 3 Primer sequence for RT qPCR of rat gingival tissue.

|  | **Primer** | | **Sequence (5’ to 3’)** |
| --- | --- | --- | --- |
| Clinical sample | OSBPL6-F | | ACTTGCTGCTCAGGAGGTAT |
|  | OSBPL6-R | | CTCGGTGCTAGAGGAAGCAG |
|  | NUDT15-F | TGGAGTTCGGTGAAACCTGG | |
|  | NUDT15-R | AAACAACGCAGTCCCCAGAA | |
|  | ENTPD1-F | TGCGGGTTCTTCTCACACAA | |
|  | ENTPD1-R | CCCAGGTAAACGGGTGTCTC | |
|  | CYP24A1-F | AGCGATAATACGCCTCAGATGG | |
|  | CYP24A1-R | GATGGTGCTGACACAGGTGA | |
|  | ADA-F | AGATGAAGGCCAAAGAGGGC | |
|  | ADA-R | GCCTTGACCCCGAAGTCTC | |
|  | TDO2-F | GCACCGAGTGTCAGTGATCC | |
|  | TDO2-R | GTCCAAGGCTGTCATCGTCT | |
|  | GAPDH-F | ATGGGCAGCCGTTAGGAAAG | |
|  | GAPDH-R | AGGAAAAGCATCACCCGGAG | |
| Rat model | ENTPD1-F | CGTGAGTACCTCTCTCGCAG | |
|  | ENTPD1-R | GGTCAGTCCCACAGCAATCA | |
|  | CYP24A1-F | CGGAACGTCACCTCCTTACC | |
|  | CYP24A1-R | TTTGATCTCTAGCCGCTGGG | |
|  | TDO2-F | TGTCATACCGTGCACTCCAG | |
|  | TDO2-R | CCTTGTACCTGTCGCTCACA | |
|  | GAPDH-F | TGTGTCCGTCGTGGATCTGA | |
|  | GAPDH-R | GAGTTGCTGTTGAAGTCGCA | |
