# Supplementary material for: Mitochondrial Metabolic Biomarkers in Periodontitis: Discovery and Clinical Validation
Source: Int Dent J. 2026 Jun 13;76(4):109682. doi: 10.1016/j.identj.2026.109682 (PMC13279168; doi:10.1016/j.identj.2026.109682)
Supplement: Supplementary file 7 [file mmc7.docx]

**Supplementary Table 4 Marker genes for cell annotation.**

| **Cell cluster** | **Cell type** | **Marker gene** |
| --- | --- | --- |
| 0 | Endothelial cell | COL15A1, SELP |
| 1 | Fibroblast | LUM, COL1A2 |
| 2 | T cell | CD3D, CD3G |
| 3 | T cell | CD3D, CD3G |
| 4 | Plasma cell | IGHA1, IGHG4 |
| 5 | Macrophage | SERPINA1, IL1B |
| 6 | Smooth muscle cell | MYH11,ACTA2,TAGLN |
| 7 | Basal cell | FXYD3, CALML3 |
| 8 | B cell | MS4A1, CD79B |
| 9 | Endothelial cell | ADAMTS6, ARL15 |
| 10 | Mast cell | CPA3, TPSAB1 |
| 11 | T cell | CD3D, CD3G |
| 12 | Fibroblast | CD14,CD68,MS4A7, |
| 13 | M2 macrophage | FABP4, PDPN |
| 14 | Fibroblast | MCAM,RGS5 |
| 15 | pDC | IRF4, PLD4 |
| 16 | Neural progenitor cell | MKI67, BIRC5 |
| 17 | Melanocyte | MITF, PMEL, TYR |
